# Otoferlin as a multirole Ca^2+^ signaling protein: from inner ear synapses to cancer pathways

**DOI:** 10.3389/fncel.2023.1197611

**Published:** 2023-07-19

**Authors:** Jean-Christophe Leclère, Didier Dulon

**Affiliations:** ^1^Department of Head and Neck Surgery, Brest University Hospital, Brest, France; ^2^Laboratory of Neurophysiologie de la Synapse Auditive, Université de Bordeaux, Bordeaux, France; ^3^Institut de l’Audition, Institut Pasteur & INSERM UA06, Paris, France

**Keywords:** ferlins, Ca^2+^ sensor, membrane fusion, auditory synaptopathy, deafness, brain synapses, dysferlinopathy, cancer

## Abstract

Humans have six members of the ferlin protein family: dysferlin, myoferlin, otoferlin, fer1L4, fer1L5, and fer1L6. These proteins share common features such as multiple Ca^2+^-binding C2 domains, FerA domains, and membrane anchoring through their single C-terminal transmembrane domain, and are believed to play a key role in calcium-triggered membrane fusion and vesicle trafficking. Otoferlin plays a crucial role in hearing and vestibular function. In this review, we will discuss how we see otoferlin working as a Ca^2+^-dependent mechanical sensor regulating synaptic vesicle fusion at the hair cell ribbon synapses. Although otoferlin is also present in the central nervous system, particularly in the cortex and amygdala, its role in brain tissues remains unknown. Mutations in the OTOF gene cause one of the most frequent genetic forms of congenital deafness, DFNB9. These mutations produce severe to profound hearing loss due to a defect in synaptic excitatory glutamatergic transmission between the inner hair cells and the nerve fibers of the auditory nerve. Gene therapy protocols that allow normal rescue expression of otoferlin in hair cells have just started and are currently in pre-clinical phase. In parallel, studies have linked ferlins to cancer through their effect on cell signaling and development, allowing tumors to form and cancer cells to adapt to a hostile environment. Modulation by mechanical forces and Ca^2+^ signaling are key determinants of the metastatic process. Although ferlins importance in cancer has not been extensively studied, data show that otoferlin expression is significantly associated with survival in specific cancer types, including clear cell and papillary cell renal carcinoma, and urothelial bladder cancer. These findings indicate a role for otoferlin in the carcinogenesis of these tumors, which requires further investigation to confirm and understand its exact role, particularly as it varies by tumor site. Targeting this protein may lead to new cancer therapies.

## 1. Introduction

**FIGURE 1:**
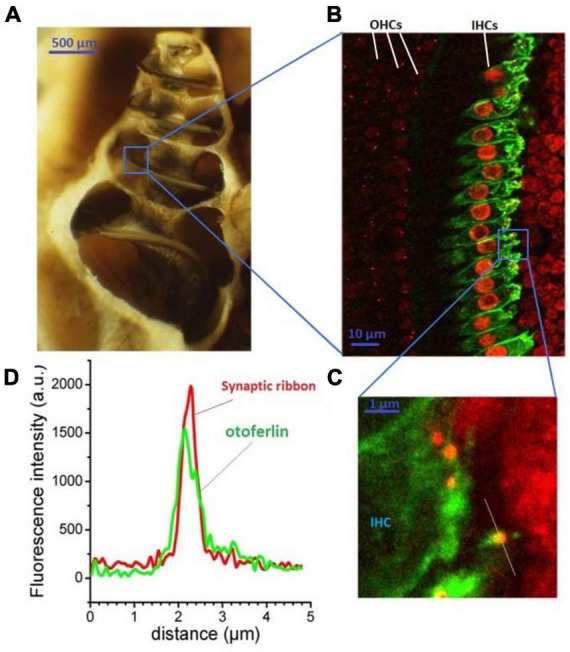
Otoferlin expression in auditory inner hair cells. The cochlea (here an open bulla from a guinea pig) is a small spiraled bone structure looking like a snail shell **(A)** that contains the hearing organ, the organ of Corti. This spiral-shaped sensory epithelium, lying on the basilar membrane between the scala vestibuli and the scala tympani, is composed of two types of hair cells: the outer hair cells (OHCs) which amplify the sound waves within the cochlea, and the inner hair cells (IHCs) which transduce the mechanical sound waves into nerve impulses. IHCs specifically express otoferlin as viewed in green under fluorescence immuno-confocal microscopy of a surface preparation of the organ of Corti **(B)**. Otoferlin is essentially expressed at the basal synaptic pole of the IHCs (at right), where the afferent auditory nerve fibers make synaptic contacts with the presynaptic IHCs ribbons [red, enlarged view in **(C)**]. An antibody against CtBP2, a constituent of the RIBEYE-B domain, is used to label the synaptic ribbons. Note that CtBP2 is also a nuclear transcription factor, explaining the additional labeling of the cell nuclei. **(D)** The presynaptic IHC ribbons are closely colocalized with otoferlin, as indicated by the line-scan fluorescence intensity profiles of the ribbon indicated in **(C)**. This is an original figure built from unpublished confocal immunostaining as previously described (Peineau et al., 2021).

**FIGURE 2:**
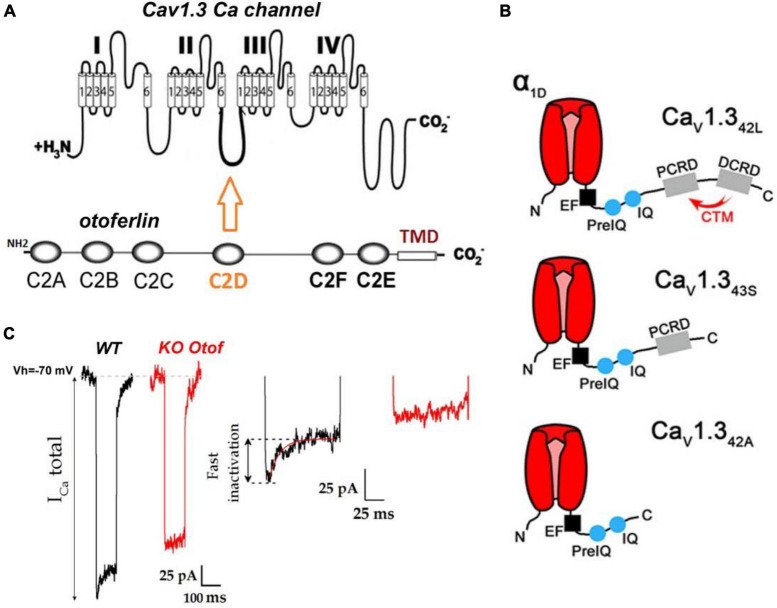
Otoferlin interaction with Ca_V_1.3 Ca^2+^ channels. **(A)** Protein-protein interaction studies have shown otoferlin interaction with the II-III loop of the Ca^2+^ channel α subunit (Ramakrishnan et al., 2009, 2014; Hams et al., 2017). **(B)** Hair cells (in particular IHCs) of the auditory organ express several C-terminal splicing isoforms of Ca_V_1.3: Ca_V_1.3_42L_, a long isoform with no or poor current inactivation, Ca_V_1.3_43S_, and Ca_V_1.3_42A_ short truncated C-terminal isoforms with fast inactivation, presumably carrying about 10% of the total inner hair cell (IHC) calcium current (Vincent et al., 2017). The absence of inactivation of the Ca_V_1.3_42L_ subunit is due to the presence at its extreme C terminus of two α helix proximal (PCRD) and distal C-terminal regulatory domains (DCRD) that interact with each other to form the C-terminal regulatory domain (CTM). This CTM domain interaction slows down the inactivation of Ca_v_1.3 channels by competing with the binding of Ca^2+^–CaM on IQ domains, (for review see Striessnig et al., 2014) (figure modified from Vincent et al., 2017). Note that CTM is absent in Ca_V_1.3_43S_, and Ca_V_1.3_42A_ explaining the fast calcium-dependent inactivation of these channels. **(C)** As a signature of this interaction between otoferlin and Ca_V_1.3 channels, and most likely Ca_V_1.3_43S_, and Ca_V_1.3_42A_, IHCs from mice lacking otoferlin display Ca^2+^ currents with no fast inactivating component (figure modified from Figure 4B of Tertrais et al., 2019).

Interest in otoferlin began with the discovery of mutations in the OTOF gene that were identified as responsible for recessive profound deafness in humans (Yasunaga et al., 1999, 2000). This deafness is called DFNB9 (for autosomal recessive deafness 9). It represents about 2% of non-syndromic prelingual deafness, i.e., without any other associated health problem, and is the most frequent cause of auditory neuropathy spectrum disorder (ANSD) (Iwasa et al., 2019). To date, about 220 mutations leading to DFNB9 deafness have been identified (Azaiez et al., 2021). Remarkably, some otoferlin mutations, notably in the C2F domain, can lead to exacerbated deafness when body temperature rises during a fever (Varga et al., 2006; Marlin et al., 2010; Matsunaga et al., 2012; Strenzke et al., 2016). This peculiar property underlines the importance of a precise functional 3D architecture of the multi-C2 protein, which is known to interact at the presynaptic active zone of the sensory hair cells with SNARE (soluble *N*-ethylmaleimide-sensitive-factor attachment protein receptor) proteins such as SNAP25 (Roux et al., 2006; Ramakrishnan et al., 2014; Hams et al., 2017; Calvet et al., 2022) and with Ca_*v*_1.3 Ca^2+^ channels (Ramakrishnan et al., 2009; Vincent et al., 2017, 2018; Table 1 and Figures 1, 2).

**TABLE 1:** The otoferlin interactome.

Identified interacting domains of otoferlin										
Interacting factors	Putative function	Otoferlin	C2A	C2B	C2C	C2D	C2E	C2F	TMD	References
Ca^2+^	Bind to Asp residues in C2 domains	✓		✓	✓	✓	✓	✓		Johnson and Chapman, 2010; Michalski et al., 2017
Syntaxin 1A	SNARE complex	✓	✓	✓		✓	✓	✓		Ramakrishnan et al., 2009, 2014
SNAP-25	SNARE complex	✓						✓		Ramakrishnan et al., 2014
Ca_V_1.3	L-type Ca^2+^ channel	✓	✓	✓		✓		✓		Ramakrishnan et al., 2014; Hams et al., 2017
CaMKIIδ	CaM kinase	✓						✓		Meese et al., 2017
Endophilin-A1	Endocytic adaptors	✓								Kroll et al., 2019
PIP2	Phospholipid binding	✓			✓			✓		Roux et al., 2006; Ramakrishnan et al., 2009; Padmanarayana et al., 2014
Myosin VI	Motor for vesicular membrane traffic	✓				✓				Heidrych et al., 2009
Golgi marker GM130	Endosome- network dynamics	✓								Heidrych et al., 2008
Rab8b GTPase	Protein transport regulator	✓								Heidrych et al., 2008
NSF	SNARE chaperone membrane fusion	✓	✓	✓		✓		✓		Selvakumar et al., 2017
Ergic2 (ER/Golgi)	Brain specific partner	✓				✓				Zak et al., 2012
Dynamin	Fast endocytosis GTPase	✓				✓	✓	✓		Tertrais et al., 2019
AP2	Clathrin adaptor protein, slow endocytosis	✓	✓	✓		✓		✓		Duncker et al., 2013; Jung et al., 2015; Selvakumar et al., 2017
Calpain	Ca^2+^ activated protease cleaving otoferlin	✓								Redpath et al., 2014
Tryptophan-rich basic protein (WRB)	TRC40 complex, transmembrane recognition complex	✓							✓	Vogl et al., 2016

Otoferlin is a multi-C2-domain transmembrane vesicular protein with mutations associated with congenital deafness. Based on the published literature, this table recapitulates the putative factors (left column) interacting with otoferlin, its specific C2 domains, or its transmembrane domain (TMD). Most of these factors interacting with otoferlin share functions with SNARE proteins or have other neurotransmission-related functions.

**FIGURE 3:**
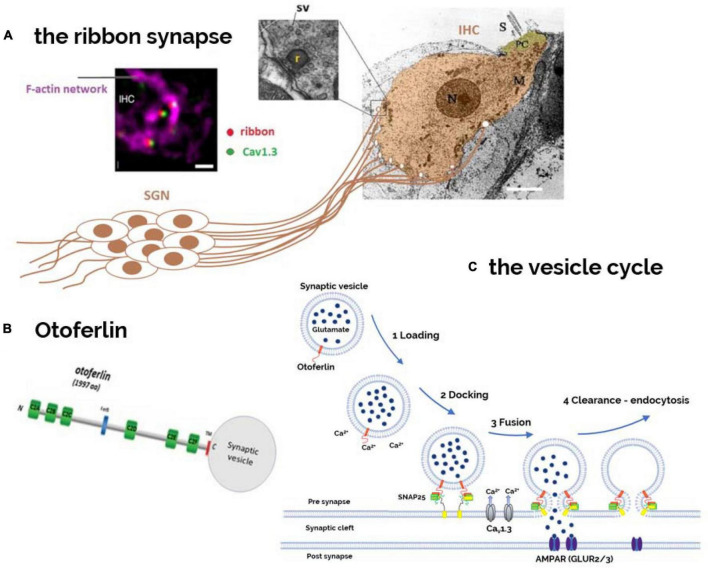
Otoferlin as a Ca^2+^-sensor controlling the hair cell synaptic vesicle cycle. **(A)** The postsynaptic structures, issued from the afferent nerve fibers of the bipolar spiral ganglion neurons (SGNs), are here schematically represented contacting the basal part of an IHC viewed under electron microscopy (EM). Each IHC is contacted by 10–20 nerve fibers, depending on their cochlear position (sound frequency encoding). Presynaptically, facing the afferent nerve boutons, is found an electron-dense 300–350 nm wide structure called ribbon to which several dozens of synaptic vesicles (SV) are attached (inset EM). Viewed under immuno-confocal fluorescence microscopy, each presynaptic ribbon (red) at the basal synaptic pole of the IHC, is attached to an F-actin cage like- structure (purple) and is closely colocalized with a single cluster of Ca_V_1.3 Ca^2+^ channels (green) (image modified from Vincent et al., 2015). **(B)** Otoferlin, is a six-C2 domain protein with a single transmembrane domain (TMD) at its C-terminal that allows its anchoring into the phospholipid membrane of synaptic vesicles and active zones of transmitter release. Otoferlin, bearing several C2-Ca^2+^ bind domains, is essential for reloading, fusion, and endocytosis of synaptic vesicles at the ribbon synapses **(C)**.

DFNB9 is caused by a defect in synaptic neurotransmission from auditory inner hair cells (IHCs) to their contacting spiral ganglion afferent fibers (Roux et al., 2006; Beurg et al., 2010; Michalski et al., 2017). The sound-induced electrical analog signal of the hair cells, also called cochlear microphonic, is incorrectly transduced into nerve impulses at the auditory nerve fibers. We recall that one of the morphological characteristics of the afferent IHC synapses is the presence of a presynaptic ribbon, an electron-dense presynaptic structure 300–350 nm wide (Figures 1, 3). Each of the 15–20 presynaptic ribbons per IHC are facing a single postsynaptic auditory nerve structure bearing AMPA (glutamate) ionotropic receptors composed of GluA2, GluA3, and GluA4 subunits (Rutherford et al., 2023). This presynaptic ribbon, composed of RIBEYE proteins arranged in staircase pattern, allows the attachment of numerous synaptic vesicles at the membrane active zone of release. This ribbon structure is essential for spike timing precision of the auditory nerve fibers (Wittig and Parsons, 2008; Becker et al., 2018; Jean et al., 2018).

Otoferlin-induced synaptopathy is due to a defective Ca^2+^-evoked exocytosis of synaptic vesicles containing glutamate, the main cochlear excitatory neurotransmitter, at the IHC ribbon synapses (Figure 3). Like in most neurosecretory cells, vesicle trafficking and synaptic exocytosis in IHCs are thought to involve interactions of SNARE complex proteins such as SNAP25 (Calvet et al., 2022). Synaptotagmins (Syts), a large family of transmembrane proteins containing tandem Ca^2+^-binding C2-domains, confer Ca^2+^ sensitivity to SNARE-dependent vesicle fusion in the CNS (central nervous system) (Chapman, 2008). However, the implication of Syts in hair cell synaptic exocytosis has been shown to be limited to immature cochlear developmental stages when hair cells fire action potentials (Beurg et al., 2010). At post-hearing mature stages, when hair cells display gradual depolarization upon sound stimulation, otoferlin becomes the major Ca^2+^ sensor triggering synaptic vesicle exocytosis.

DFNB9 is classified as a neuropathy and, more specifically, as an auditory cochlear synaptopathy (Moser and Starr, 2016). In addition to the cochlea, otoferlin expression has also been shown in the brain with unknown function (Schug et al., 2006) and, in the peripheral vestibular organs where hair cell Ca^2+^-dependent fast synaptic vesicle exocytosis is also impaired (Dulon et al., 2009). In DFNB9 patients, it is important to note that the active electromechanical sound amplification function by the outer hair cells (OHCs) is normal, as indicated by measurements of the distortion products of otoacoustic emissions (DPOAEs). The DFNB9 auditory cochlear synaptopathy is currently overlooked in clinics when the systematic hearing screening performed at birth relies only on DPOAE measurements. Since delays in the management of sensory pathology reduce the quality of language development, it is becoming essential to screen for deafness with automated auditory brainstem recordings (ABRs). For DFBNB9 hearing loss, hearing aids or cochlear implantation are the only effective treatments currently available. Future clinical gene therapies are on the way, as demonstrated by successful rescue viral gene therapies in otoferlin-deficient mouse models (Al-Moyed et al., 2019; Akil, 2020; Rankovic et al., 2020). However, in these studies, for unknown reasons, there was only a partial rescue of ABR wave I amplitude (the neural response associated with the electrical activity of the IHC ribbon synapses), suggesting a non-restored loss of some ribbon synapses. This incomplete rescuing is likely due to the late developmental stage (postnatal days P1 to P3 in mice) at which AAV treatment was performed in these studies. Indeed, otoferlin seems essential for normal early prenatal development and the maintenance of ribbon synapses (Stalmann et al., 2021).

## 2. Otoferlin structure and isoforms

**FIGURE 4:**
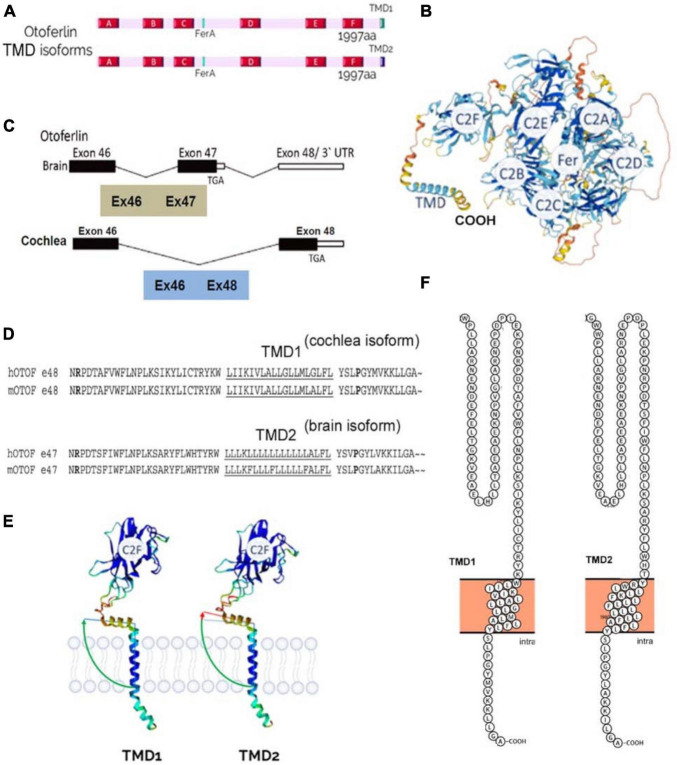
Otoferlin structure. **(A)** C2-domain structures of the two transmembrane domain (TMD) isoforms of otoferlin, TMD1 (cochlea) and TMD2 (brain). **(B)** AlphaFold prediction of otoferlin 3D-structure as a globular protein with six C2 domains (C2A-F), a Fer domain, and a C-ter TMD (structure built from AlphaFold Protein Structure Database). **(C)** Brain and inner ear tissues have been reported to express two different specific alternatively spliced forms of the otoferlin TMD, encoded by either exon-47 or exon-48, respectively (Fig adapted from Yasunaga et al., 2000). Exon-47 is skipped in the cochlea, leading to a different carboxy-terminal peptide sequence from that of the brain isoform, which expresses exon-47. In mRNA with exon-47 (neuronal otoferlin), translation terminates in that exon, leaving exon-48 untranslated. **(D)** Exon-47 of the brain isoform and exon-48 of the cochlear isoform specify the C-terminal TMD 60 amino acids of otoferlin. The amino acid sequences of the two isoforms differ essentially in the predicted transmembrane sequence (underlined). **(E)** Analysis of protein structure with AlphaFold software online (developed by DeepMind and EMBL-EBI) predicts a different angle of insertion of cochlear TMD1 and neuronal TMD2 otoferlin isoforms within phospholipid membranes. The use of a specific TMD is likely important for targeting the protein to specific subcellular locations, such as particular glutamatergic excitatory presynaptic active zones, and/or for specifying the interaction with ligands. **(F)** TMD structure analysis and representation using Protter software (Omasits et al., 2014) predicts a 21 aa TMD1 for the cochlear isoform (gi| 154240702| ref| NP_001093865.1| otoferlin [Mus musculus]) and a 23 aa for the brain isoform (gi| 154240679| ref| NP_114081.2| otoferlin [Mus musculus]).

Otoferlin is a large 1997 amino acid (aa) protein, that includes a single C-terminal transmembrane domain (TMD) anchoring the protein to the vesicular membrane and six C2 domains (A–F) oriented toward the IHC cytosol (Figures 3, 4). The C2 domains are structures composed of eight antiparallel β-sheets and negatively charged top loops due to the presence of five aspartate residues, constituting the putative binding site for Ca2 + ions (Sutton et al., 1995; Xue et al., 2008). The C2-A domain is the only C2 domain predicted to be unable to bind Ca^2+^ due to a shorter top loop, thus lacking aspartates coordinating calcium binding (Helfmann et al., 2011). The main function of C2 domains is to target membrane surfaces following Ca^2+^-binding. C2 domains bind to their target membranes by using a combination of hydrophobic and electrostatic interactions, preferentially with phosphatidylinositol 4,5-bisphosphate (PIP2) or phosphatidylserine (PS) (Corbalan-Garcia and Gómez-Fernández, 2014). Otoferlin also has a FerA domain, a four-helix bundle fold with its own Ca^2+^-dependent phospholipid-binding activity, suggesting that the interaction of this domain with the membrane is enhanced by the presence of Ca^2+^ (Harsini et al., 2018).

Otoferlin is produced mainly in the brain and inner ear (cochlea and vestibule), and in smaller amounts in other organs: the heart, liver, pancreas, kidney, and skeletal muscle (Yasunaga et al., 2000). Otoferlin appears as a globular protein when using the AlphaFold protein structure prediction software (Jumper et al., 2021; Figure 4). In humans, a long and short isoform of otoferlin have been identified (Yasunaga et al., 1999, 2000), with the long one comprising all six C2 domains and a C-terminal *trans*-membrane domain (TMD) (1997 aa), while the short one expresses only the last three C-terminal C2 domains (C2-DEF) with the TMD (1230 aa). The role of these two isoforms remains unknown. Of note, the short otoferlin isoform is not present in mice, whereas the human long isoform has 98% sequence similarity with murine otoferlin.

The long isoform of otoferlin is encoded by 48 exons, of which exons 6 and 31 can have alternative splicing. Exon 47 also carries an alternative splice site, and upon splicing, exon 48 is expressed and encodes a transmembrane domain called TMD1. If exon 47 is not spliced, it bears a stop codon at its end, so the translation of exon 48 is excluded, and otoferlin has a different TMD domain, TMD2 (Figure 4). In humans and mice, the TMD2 otoferlin isoform (exon 47) is preferentially expressed in the brain, while the TMD1 isoform (exon 48) is specifically found in the cochlea (Yasunaga et al., 2000; Schug et al., 2006). Specific mutations in exon 48 cause hearing loss (Choi et al., 2009), underscoring the importance of TMD. According to Alphafold3D structure predictions, the differences between the two TMD isoforms are partly based on the angulation of the transmembrane domain relative to the rest of the protein (Figure 4E). These two TMDs only share less than 57% amino acid sequence similarity (Figure 4D). Notably, the brain TMD2 isoform contains a large peptidic sequence with numerous contiguous Leu residues (Leu-block of more than 9 Leu) in comparison to the cochlear TMD isoform, which contains discontinuous stretches of Leu residues (Figure 4D). Large hydrophobic Leu-blocks in TMD peptides confer greater helicity and circumferential hydrophobicity that facilitate biological membrane insertion (Stone et al., 2015). The use of specific TMD should be important for targeting the protein to specific subcellular locations, such as particular glutamatergic excitatory presynaptic active zones, and specifying the interaction with specific organelles and ligands (Sharpe et al., 2010). Mutations in exon 48, such as the in-frame deletion of a conserved isoleucine in the cochlear TMD1 at position 1967 (p.Ile1967del), lead to hearing impairment and underline the importance of this domain in targeting the protein to the endoplasmic reticulum (ER) membrane in hair cells (Vogl et al., 2016).

Another isoform, with an alternative splice in exon 31 might explain deafness caused by heat sensitivity, but its presence in the human cochlea remains uncertain (Strenzke et al., 2016). A recent study, by Liu et al. (2023) provides new insights into the role of alternative otoferlin isoforms in auditory function and their modulation by environmental factors such as noise and aging. This study discovered a new short transcript of otoferlin derived from an unannotated exon 6b whose expression is increased in IHCs encoding high frequency sounds and varies under noise and aging conditions.

## 3. Genetic mutations and DFNB9

**FIGURE 5:**
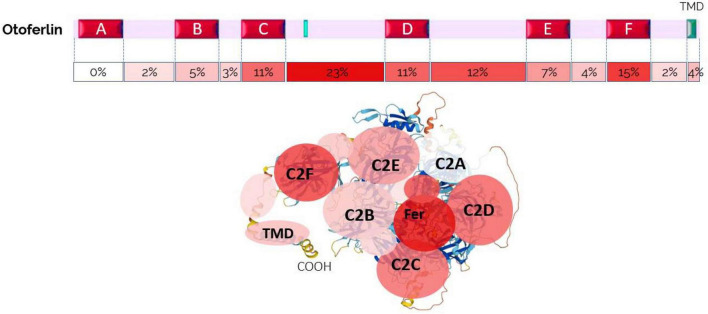
Frequency of OTOF pathogenic mutations according to their location in the different domains of Otoferlin, showing the particular importance of the C, D region and the F domain (map established from the Human Gene Mutation Database).

Approximately 220 mutations causing DFNB9-type deafness have been identified, some of which cause deafness during a febrile episode (Vona et al., 2020; Azaiez et al., 2021). Interestingly, there is no described pathological mutation of the C2A domain (Figure 5; Stenson et al., 2003). All other areas of the protein can be affected. According to the Human Gene Mutation Database, 41% of the variants are missense, 22% nonsense, 15% deletions, 14% splicing, 6% insertions, and 2% copy number variants. The missense variants alter the folding, stability, or function of the protein. Truncating variants (nonsense, deletion, splicing, insertion, and copy number variation) result in a missing or truncated non-functional protein. Both types of variants result in severe to profound hearing loss. One of the most common OTOF mutations (p.R1939Q) is located at the junction between the calcium binding C2F domains and the C-terminal TMD (Matsunaga et al., 2012; Kim et al., 2018). As mentioned above, some of the mutations lead to a disorder of the temperature-sensitive auditory neuropathy spectrum. Elevated body temperature causes severe to profound hearing loss, whereas normal body temperature results in normal to moderate hearing. Interestingly, all identified thermosensitive mutations are located between the C2C and C2F domains, underscoring the functional importance of these regions (Zhu et al., 2021).

The mechanisms underlying the temperature sensitive hearing loss remain essentially unknown, and they may vary with their site of location. These mutations probably alter the folding or unfolding properties of the long multi-C2-domain protein, impairing specific Ca^2+^-dependent C2-domain interactions and synaptic vesicle fusion, as temperature rises. Another hypothesis is that there is increased degradation and loss of the protein. This is caused by the heat sensitivity of otoferlin, especially in the presence of variants such as Ile515Thr (Strenzke et al., 2016). The protein is then degraded more quickly.

## 4. Otoferlin tissue expression

**FIGURE 6:**
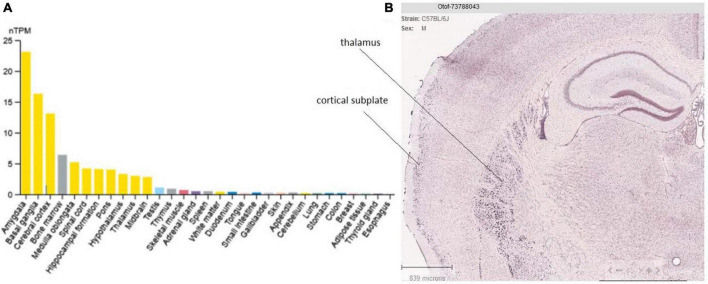
Tissue and brain expression of otoferlin. **(A)** Tissue specificity of otoferlin RNA expression (data from the Human Protein Atlas). **(B)** Otoferlin expression (mRNA *in situ* hybridization) in the mouse brain thalamus and cortex (cortical subplate) from the Allen Brain Atlas portal. This analysis appears to well corroborate previous findings showing otoferlin expression in brain tissues (Schug et al., 2006).

The tissue distribution of otoferlin is primarily observed in the brain and inner ear (Schug et al., 2006). In the brain it is mainly found in the amygdala and cerebral cortex (Figure 6). We recall that the two TMD otoferlin isoforms have different distributions: one is almost exclusively found in the cochlea (TMD1, exon 48), and the other is mainly found in the brain (TMD2, exon 47) (Yasunaga et al., 2000). However, this TMD variant distribution in the brain and inner ear, only demonstrated by RT-PCR and Northern blot analysis at the tissue level, needs to be confirmed at the protein and cellular levels.

Otoferlin expression increases in hair cells as they mature, remaining permanently expressed in IHCs and only transiently expressed in immature OHCs (largely decreasing after P4-P6 in mice) (Roux et al., 2006; Schug et al., 2006). This transient expression in OHCs is essential for spontaneous synaptic exocytosis by immature OHCs, a process likely involved in the establishment of frequency tonotopy of the central auditory nuclei during development (Beurg et al., 2008). In IHCs and vestibular cells, otoferlin was localized in synaptic vesicles by immunogold labeling (Roux et al., 2006; Dulon et al., 2009) or in the Golgi apparatus and presynaptic zone by immunohistochemistry (Schug et al., 2006; Heidrych et al., 2008). In HEK cell expression, otoferlin is found at the plasma membrane and colocalizes with some of the *trans*-Golgi markers (GM130 or TGLON2) (Redpath et al., 2016). In IHCs, otoferlin is distributed throughout the cytoplasm and plasma membrane, except for the apical portion that forms the cuticular plate and tight junctions with neighboring cells. It is also interesting to note that the expression of the protein differs according to its two isoforms, TMD1 and TMD2, when expressed in heterologous cell lines. In HEK293 cell cultures, TMD1 showed a dispersed pattern within the cell, while TMD2 was predominantly found at the plasma membrane (Redpath et al., 2016). Otoferlin is also expressed by type I and type II vestibular utricular hair cells (Dulon et al., 2009). Note that truncation of the TMD of otoferlin alters the development of hair cells and reduces membrane docking (Manchanda et al., 2021). This tail-anchored protein can be inserted into the endoplasmic reticulum (ER) of hair cells via the TRC40 receptor tryptophan-rich basic protein (Wrb) (Vogl et al., 2016).

## 5. Otoferlin functions and mechanisms of action

### 5.1. Otoferlin multirole at the IHC synaptic vesicle cycle

Remarkably, mature inner hair cells (IHCs) lack the Ca^2+^-sensors synaptotagmin (I and II) and Munc13, which are known to play a crucial role in vesicle exocytosis at conventional central neuronal synapses (Beurg et al., 2010; Reisinger et al., 2011; Vogl et al., 2015). It is worth recalling that mature IHCs, contrary to central neurons, do not fire action potentials but are capable responding to a large dynamic range of sound intensity (>100 dB) by graded depolarization that is encoded into an increasing discharge rate at the postsynaptic nerve fibers. It is now established that otoferlin plays a role at several essential stages of the vesicle cycle in IHCs, including functional docking, priming, fusion, endocytosis, and possibly transport and maturation of vesicles (Figure 3; Michalski et al., 2017; Tertrais et al., 2019). In otoferlin knockout mice, the average tether length between vesicles and the active zone is increased, suggesting a possible defect in vesicle attachment and priming (Vogl et al., 2015). The downstream calcium-triggered exocytosis of vesicles is almost completely blocked in otoferlin knockout mice. Otoferlin is also involved in active zone clearance, including the transport of exocytic material, through interactions with adaptor protein 2 μ, a motor protein, and a GTPase (Jung et al., 2015). Vesicle size may indicate a role for otoferlin in vesicle reformation and maturation, and the functions of otoferlin may be regulated by phosphorylation.

### 5.2. Otoferlin and membrane fusion

Structural analysis and prediction using Protter (Omasits et al., 2014) and Alphafold (Jumper et al., 2021) display otoferlin as a globular protein anchored to the vesicle and/or plasma membrane through its C-terminal domain, while its N-ter Ca^2+^-sensing C2-domains are in the cytosol (Figure 4). The structural features of its C2-domains, through their Ca^2+^-binding and phospholipid-binding activities, allow otoferlin to play a role in membrane fusion and trafficking, in endocytic, secretory, and lysosomal pathways. By deleting exons 14 and 15 of the OTOF gene, an OTOF KO mouse model was created (Roux et al., 2006). These otoferlin-deficient mice (*Otof ^–/–^*) are profoundly deaf. Patch-clamp recordings for time-resolved change of membrane capacitance in IHCs from *Otof ^–/–^* mice show a significant impairment of fast and sustained exocytotic activity (Beurg et al., 2010; Michalski et al., 2017). Another mouse model, Pachanga, carrying a missense mutation in the C2-F domain of otoferlin, was obtained by ENU (N-ethyl-N-nitrosourea) mutagenesis (Schwander et al., 2007). Pachanga mice have profound hearing loss, but unlike *Otof ^–/–^* mice, IHCs still weakly express otoferlin and maintain rapid fusion of the pool of fusogenic vesicles, forming the RRP (for “readily releasable pool of vesicles”) (Pangrsic et al., 2010). However, IHC exocytosis cannot be sustained upon repeated stimulations in these Pachanga mutant mice, indicating impaired recruitment of vesicles to IHC fusion sites. These results suggested that otoferlin is also an essential calcium sensor for synaptic vesicle replenishment at IHC active zones. It should be noted that vesicle recruitment is a calcium-dependent process in hair cells (Spassova et al., 2004; Levic et al., 2011). Gradual intracellular Ca^2+^-uncaging experiments in IHCs revealed that otoferlin-induced exocytosis has an intrinsically high Ca^2+^ sensitivity (affinity) with a mean Kd of 4.0 ± 0.7 μM and responses spanning from 1 to 20 μM Ca^2+^ (Vincent et al., 2014). As a comparison, the neuronal Ca^2+^ sensors, synaptotagmins, have nearly a one-order of magnitude lower Ca^2+^ affinity (higher Kd), a property in good correlation with their specific functions of being activated only by high Ca^2+^ concentrations typical for AP-evoked Ca^2+^-elevations near Ca^2+^ channels (Bornschein and Schmidt, 2018).

On the other hand, the importance of otoferlin in the maintenance or development of ribbons and the presence of corresponding synapses has been demonstrated in mice (Stalmann et al., 2021). Otoferlin depletion in zebrafish hair cells also results in abnormal synaptic ribbons and altered intracellular calcium levels (Manchanda et al., 2019), suggesting Ca^2+^ as an important factor for the maintenance and survival of the ribbons as in aging auditory synapses (Peineau et al., 2021).

Although some of the interacting proteins start to be identified (Table 1), the precise mode of action of otoferlin in membrane fusion is not fully elucidated, particularly in comparison with the neuronal calcium sensor (synaptotagmin1) which regulates exocytosis function as a multimer with oligomerization via a cluster of juxtamembrane linker (Courtney et al., 2021). Note that, in contrast to ferlin proteins, the TMD of synaptotagmins is located at the N-terminal region of the protein for unknown reasons. The homolog of otoferlin, dysferlin, has also been shown to dimerize in living cells, at its transmembrane domain and at its multiple C2 domains (except C2A), as probed by fluorescence resonance energy transfer (FRET) (Xu et al., 2011).

The C2-A domain is the only one of the six otoferlin domains that cannot bind Ca^2+^, which probably explains the absence of pathogenic mutations in this domain (Johnson and Chapman, 2010; Helfmann et al., 2011; Padmanarayana et al., 2014). The idea of a simple repeat between each C2 domain is rejected. An *Otof* KI mouse model carrying two mutations in C2C (substitution of 2 aspartic acid by alanine, affecting Ca^2+^ binding), demonstrated that otoferlin is essential in both rapid fusion and vesicle recruitment (Michalski et al., 2017). Interestingly, the viral expression of truncated forms of otoferlin (C2-EF, C2-DEF, and C2-ACEF) can only partially rescue the fast and transient release component of exocytosis in mouse hair cells lacking otoferlin (*Otof ^–/–^*), yet cannot sustain exocytosis after long, repeated stimulations (Tertrais et al., 2019). To note, a C2-EF otoferlin truncated form can also be produced by intracellular calpain-1 digestion in cell lines (Redpath et al., 2014) but it is unknown whether such truncation occurs in auditory IHCs.

Note that otoferlin is also essential for the fast exocytosis of type I vestibular cells. *Otof*^–/–^ mice show altered vestibular compound action potentials, suggesting impaired vestibular hair cell function (Dulon et al., 2009). In intact utricles *ex vivo*, otoferlin was found to be critical for a highly sensitive and linear calcium-dependent exocytosis, facilitating the linear encoding of low-intensity stimuli at the vestibular hair cell synapse. Surprisingly, mice and humans lacking otoferlin do not have apparent vestibular symptoms. The lack of vestibular phenotype may be explained by some compensatory mechanisms, in particular by stimulus-evoked acidification of the synaptic cleft of vestibular hair cells (protons acting as neurotransmitters) (Highstein et al., 2014) or by the use of another type of neurotransmission via potassium accumulation in the calyx, which depolarizes it (Holt et al., 2007). Although these compensatory modes of transmission are rather slow and non-linear, they may explain the absence of a vestibular phenotype in OTOF patients and animal models.

### 5.3. A specific mechanical tuning between Ca^2+^, otoferlin, F-actin, and the ribbon could determine the specific firing frequency of each auditory nerve fiber

**FIGURE 7:**
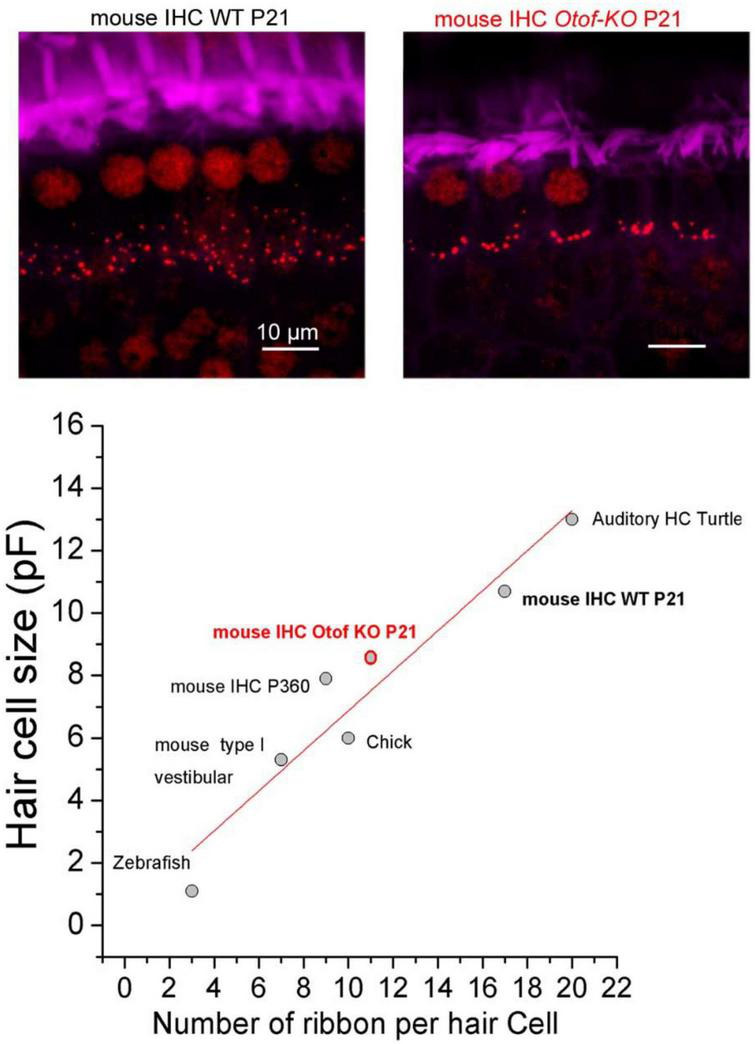
Positive linear correlation between hair cell size and ribbon number per hair cell. The two upper confocal images illustrate the comparative distribution and number of synaptic ribbons within a row of 6 IHCs from P21 Wild-Type (WT) and *Otof-KO* mice (red; immunolabeling against CtBP2, a transcriptional repressor identical to the B domain of RIBEYE, a main constituent of ribbon structure, labeling both the nucleus and the synaptic ribbons). The stereocilia of the IHCs are labeled with fluorescent FITC-phalloidin (purple). The graph was built by using data obtained from turtle auditory hair cells (low frequency coding; Schnee et al., 2011), mouse IHC WT and KO from Vincent et al. (2014), mouse IHC from old P360 mice from Peineau et al. (2021), chick hair cells from Levic et al. (2011), mouse type I vestibular hair cells from Vincent et al. (2014), zebrafish hair cells from Ricci et al. (2013) and Graydon et al. (2017). The resting size of the hair cells was measured using whole-cell patch clamp recordings of electrical capacitance in absence of stimulation. Data were well fitted with a linear correlation (*R*^2^ = 0.91). Note that hair cells of *Otof-KO* mice have a decreased number of synaptic ribbons and proportionally smaller cell sizes as compared to WT mice (Vincent et al., 2014, 2017; Tertrais et al., 2019), suggesting otoferlin as essential for the maintenance of both ribbons and cell sizes, possibly by regulating intracellular hydrostatic pressure and the F-actin membrane network (Vincent et al., 2015).

**FIGURE 8:**
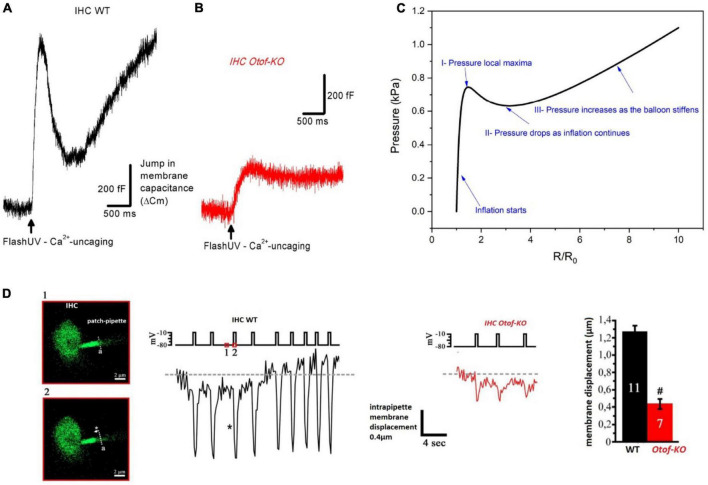
Modeling IHC as a hyper-elastic structure whose changes in membrane capacitance upon intracellular Ca^2+^ uncaging and otoferlin action behave like the pressure change in an inflating rubber balloon. **(A)** Typical example of membrane capacitance change upon intracellular Ca^2+^ uncaging in an IHC of a WT P20 mouse. **(B)** Similar experimental conditions in an IHC of a P20 *Otof-KO* mouse. In **(A,B)**, IHCs were whole-cell voltage-clamped at –70 mV and loaded with the caged-Ca^2+^ molecule DM-nitrophen [figures **(A,B)** are unpublished traces obtained as described in Vincent et al. (2014, 2015) and Tertrais et al. (2019)]. An increase in membrane capacitance reflects the fast fusion of synaptic vesicles at the plasma membrane. Note that the large N-shaped curve of the change in membrane capacitance in WT IHC is absent in IHC *Otof-KO*. **(C)** Pressure variation inside an inflating rubber balloon displays a similar N-shaped curve over time, or radius change, with the following relationship: $P=K\,\left[\frac{R_{0}}{R}-{\left(\frac{R_{0}}{R}\right)}^{7}\right]\,\left[1+0.1\,{\left(\frac{R}{R_{0}}\right)}^{2}\right]$ where R is the radius, R0 is the initial radius, K is a constant dependent on material properties, and P is the internal pressure of the balloon (Müller and Struchtrup, 2002; Wang et al., 2018; Ajmal et al., 2023). **(D)** In whole-cell configuration, IHCs were loaded with the fluorescent Ca^2+^ indicator molecule OGB-2 and the edge of ruptured plasma membrane inside the patch pipette could be visualized under confocal fluorescence microscopy. As evidence of intracellular pressure changes in IHCs, upon repeated depolarizing pulses, fast intrapipette membrane displacements could be visualized and measured in WT IHCs. These membrane movements are largely reduced in IHCs lacking otoferlin (figure modified from Vincent, 2015). ^#^indicates statistical difference, unpaired *t*-test with *p* < 0.01. Overall, these elements suggest otoferlin as a mechanical protein whose interaction with Ca^2+^ leads to the development of mechanical forces that change intracellular pressure in IHCs. The changes in intracellular pressure could result from Ca^2+^-induced fast conformational changes of otoferlin and/or the fast, massive, addition of vesicular membrane to the plasma membrane.

**FIGURE 9:**
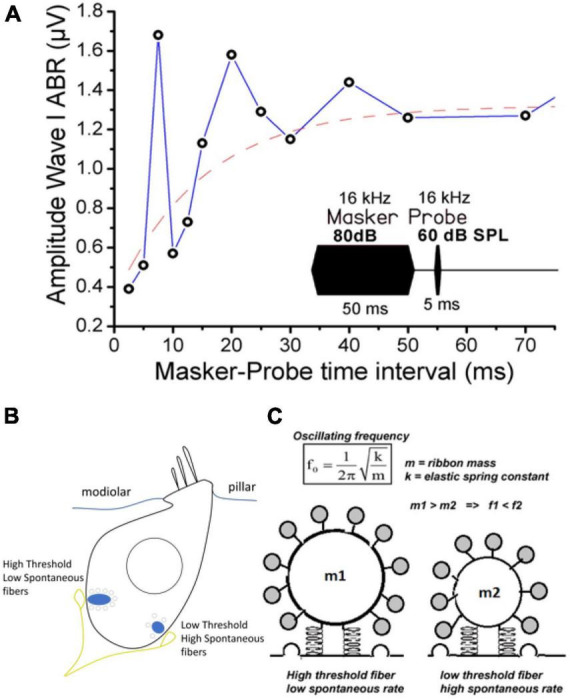
Modeling the IHC synaptic ribbon as a pulsating microsphere at the origin of signal overshoot. **(A)** Tone-on-tone forward masking: typical recovery timing from cochlear synaptic depression (ABR Wave I amplitude) in a WT mouse when using a forward masking paradigm with increasing time intervals. Note that the overshoot-like responses in the amplitude of the probe-evoked ABR amplitude (also described in gerbils and guinea-pigs, Chatterjee and Smith, 1993; Haddadzade et al., 2021) mimick the overshoot responses described in psychoacoustic studies (Zwicker, 1965; Bacon and Moore, 1987; Bacon, 1990). Although it could reflect in part the compressive input-output characteristic of the basilar membrane, we propose that the damped oscillatory behavior of the Wave I amplitude can be explained by a persistent long-lasting pulsating synaptic activity between hair cells and auditory nerve fibers at the end of the masker stimulation. The red dashed line fits with a single exponential the overall masking recovery of the auditory nerve responses with a time constant of 20 ms, a similar time scale reported for the Ca^2+^-otoferlin-dependent process to refill the hair cell ribbons with synaptic vesicles (Spassova et al., 2004; Levic et al., 2011). **(B)** Schematic drawing of an IHC depicting the two main types of postsynaptic afferent fibers regarding their spontaneous activity and threshold response. For clarity only two fibers were depicted. Note that high threshold fibers with low spontaneous activity, contacting large ribbons, are mainly located at the neural (modiolar side) of the IHCs, while low threshold fibers are at the pillar side (Liberman, 1982; Liberman et al., 2011). **(C)** Hypothetical representation of two pulsating ribbons whose mechanical oscillatory frequency will essentially depend on their mass factor as a spring-block oscillator model. The oscillatory mechanical waves likely originate from the interaction of Ca^2+^-otoferlin and the membranous F-actin network (not represented). Small ribbons are predicted to generate a higher oscillating frequency as compared to large ones.

Mammalian auditory inner hair cell (IHC) ribbon synapses have to deal with the great challenge of encoding an extremely wide range of sound intensities, with a dynamic range of more than 100 dB. To perform this challenging task, IHCs have partitioned their synaptic output sensitivity and dynamic range. Indeed, each IHC forms synapses with a pool of 10 to 30 afferent nerve fibers, among which spontaneous activity, acoustic threshold, and dynamic range vary widely. One of the most important questions in auditory neuroscience is to elucidate the functional mechanisms that dictate the synaptic diversity of each type of sensory synapses within a single IHC. Variation in the voltage-gating and spatial organization of Ca_*V*_1.3 calcium channels at each presynaptic active zone has been proposed to determine the firing specificity of the auditory nerve fibers (Özçete and Moser, 2021). Other factors, such as a mechanical tuning of each ribbon, could also participate in the firing frequency characteristic of each auditory fiber (Figures 7–9).

Otoferlin has been proposed to control IHC intracellular hydrostatic pressure and in turn exocytosis, presumably via interactions with a synaptic F-actin network (Vincent et al., 2015) and the Ca^2+^ channels Ca_*V*_1.3 at the ribbon (Vincent et al., 2017). Otoferlin is essential for synchronous multivesicular release at IHC active zones; this process underlying fast transient H^+^-inhibition of Ca_*V*_1.3 Ca^2+^ channels (Vincent et al., 2018).

Remarkably, there is a good linear correlation between the size of the hair cells and their number of synaptic ribbons per cell when comparing the ears of various vertebrates (Figure 7). In IHCs from mice lacking otoferlin, the number of synaptic ribbons is proportionally reduced with the cell size (Tertrais et al., 2019). Also in old P360 C57BL/6J mice, the reduced size of the IHCs is proportionally associated with a lower number of synaptic ribbons (Peineau et al., 2021). This correlation suggests that the number of ribbons may influence membrane exocytosis, and in turn, cell size, possibly by modifying the intracellular hydrostatic pressure. Numerous studies in various cell type have shown a reciprocal link between cell biomechanics, the submembrane actomyosin network, and exocytosis (Wang and Galli, 2018). Auditory IHCs seem to be able to sense the biomechanical properties of the environment since their exocytotic properties are sensitive to changes in intracellular hydrostatic pressure (Vincent et al., 2014). Membrane addition during hair cell synaptic exocytosis can be visualized by confocal-fluorescent microscopy (Hudspeth and Issa, 1996), indicating membrane deformation and a possible local change in hydrostatic pressure.

We propose that otoferlin, by controlling membrane fusion, regulates plasma membrane rigidity and tension of its underlying actomyosin meshwork, whose mechanical properties are likely essential to maintaining active membranous synaptic ribbons. Note that upon UV-flash-photolysis allowing ultrafast intracellular Ca^2+^ uncaging from DM-nitrophen, the change in IHC membrane capacitance (produced by massive vesicle fusion to the plasma membrane) behaves like an inflating rubber balloon (Figures 8A–C), suggesting a non-monotonic relation (non-linear stress-strain response) between the elasticity of the IHC plasma membrane and the intracellular pressure. Changes in IHC intracellular pressure can directly be visualized during exocytosis when monitoring membrane displacement during repetitive depolarizing steps (Figure 8D; Vincent, 2015). It is tempting to propose the synaptic ribbons as pulsating microspheres that are activated by Ca^2+^-otoferlin interactions with the membranous F-actin network; these mechanical interactions being the source generator (hydrostatic force) triggering vesicle membrane fusion. In this model, the mechanical oscillatory frequency of the ribbons would essentially depend on their mass, similarly to a spring-block oscillator, a factor that would then determine the firing frequency of the postsynaptic afferent nerve fibers (Figure 9). In this context, it is interesting to note that amphibian hair cells have been shown to display frequency selectivity in synaptic exocytosis, presumably due to a tonotopic variation of Ca^2+^ buffers (parvalbumin 3 and calbindin-28K) that tunes a regime of spontaneously oscillatory vesicle release (Patel et al., 2012).

### 5.4. The ribbon synapse: an unconventional synapse

Intriguingly, some proteins usually present in glutamatergic synapses of the central nervous system, such as synaptophysin, synaptotagmin, and complexins, are absent from IHC synapses, suggesting that an unconventional mechanism of neurotransmitter release may be involved (Safieddine and Wenthold, 1999; Uthaiah and Hudspeth, 2010; Nouvian et al., 2011). However, some classical neuronal SNARE proteins, such as SNAP-25, are essential for normal exocytotic function of the IHC ribbon synapses (Calvet et al., 2022). Also, molecular interactions of otoferlin with Soluble NSF Attachment Protein Receptor (SNARE) proteins, such as syntaxin-1 and SNAP-25, but not synaptobrevin-1 (VAMP-1) have been shown in protein-protein interaction studies *in vitro* (Ramakrishnan et al., 2014; Hams et al., 2017; Table 1). At the IHC synapse level, mutations in genes encoding proteins other than otoferlin are also implicated in auditory synaptopathies leading to congenital deafness vesicular glutamate transporter (VGLUT3) (Ruel et al., 2008), L-type voltage-dependent Ca^2+^ channel Ca_*V*_1.3 (CACNA1D) (Platzer et al., 2000; Baig et al., 2011), and Usher proteins such as harmonin (Gregory et al., 2011) and the tetraspan protein clarin-1 (Dulon et al., 2018).

## 6. Ferlin family

Otoferlin belongs to the ferlin protein family, which in humans has five members in addition to otoferlin (fer1L2): dysferlin (fer1L1), myoferlin (fer1L3), fer1L4, fer1L5, and fer1L6 (Lek et al., 2010; Redpath et al., 2016). The first ferlin identified was Fer-1, in Caenorhabditis elegans. Fer-1 is a fertilization factor required for the fusion of specialized vesicles with the plasma membrane during spermatogenesis (Achanzar and Ward, 1997). The main common features of ferlins are:

• The presence of multiple C2 domains that share a high sequence similarity of about 100 amino acids, and most often allow binding to Ca^2+^, phosphatidylinositol 4,5-bisphosphate (PIP2) or phosphatidylserine (PS).
• The presence of “FerA” domains also allows binding to phospholipid membrane.
• Their anchoring to the membrane by a single C-terminal domain (tail-anchored proteins).

Their common role would therefore concern Ca^2+^-triggered membrane fusion and trafficking, in endocytic, secretory, and lysosomal pathways (McNeil and Kirchhausen, 2005; Jiménez and Bashir, 2007; Lek et al., 2012). Dysferlin and myoferlin mutations cause muscle diseases: limb-girdle muscular dystrophy type 2B (LGMD2B), Miyoshi myopathy (Dysferlin), and muscular dystrophy with cardiomyopathy (Myoferlin) (Bashir et al., 1998; Liu et al., 1998).

**FIGURE 10:**
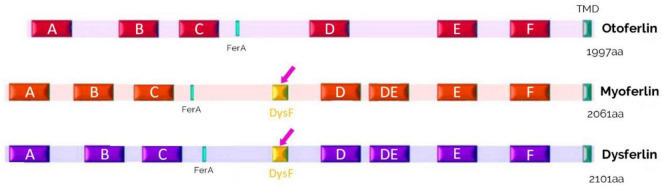
Schematic representation of the structures of otoferlin, myoferlin and dysferlin. These proteins of the ferlin family contain 6 to 7 C2 domains, a FerA domain, and a single C-terminal transmembrane domain (TMD). A domain called dysferlin (DysF) is located between the C2-C and C2-D domains in myoferlin and dysferlin (pink arrows).

Dysferlin (Fer1L1) is ubiquitously expressed, with high expression in the brain, heart and skeletal muscle (Anderson et al., 1999). Dysferlin is composed of seven C2 domains, the DysF domain and the FerA domain preceding the FerB domain (Figure 10). Dysferlin acts as a primary emergency regulator of membrane repair in a calcium-dependent manner following membrane damage to myofibres (Bansal et al., 2003; Barthélémy et al., 2018). Dysferlin is also involved in Ca^2+^ metabolism regulation in injured muscle fibers (Hofhuis et al., 2017). Dysferlin can be cleaved by calpain (1 and 2; calcium-dependent protease) in response to calcium influx following a membrane micro-lesion (Lek et al., 2013). Cleavage releases a “mini-dysferlin-C72” (with C2E, C2F, and TMD). Mini-dysferlin-C72 is selectively recruited and is thought to be a minimal configuration required for physiological membrane repair function (Krahn et al., 2010; Lek et al., 2013; Redpath et al., 2014). Otoferlin and myoferlin can also release a module with the last two C-terminal domains and the TMD after enzymatic cleavage of calpain *in vitro* (Redpath et al., 2014; Piper et al., 2017). This structure carries a high structural similarity to the synaptotagmin family. Moreover, a phylogenetic study reveals that these two C-terminal C2 domains are the most evolutionarily conserved domains of the ferlin family: there is 90% sequence similarity of C2-EF domains between mammals and mollusks, suggesting a key function (Lek et al., 2010).

Like otoferlin, the C2 domains of dysferlin bind to phosphatidylserine (PS), and phosphatidylinositol 4,5-bisphosphate in a Ca^2+^-dependent fashion (Therrien et al., 2009). Dysferlin interacts with caveolin 3 and MG53, which play an important role in maintaining dysferlin within the plasma membrane and enabling effective muscle membrane repair (Matsuda et al., 2001; Hernández-Deviez et al., 2008; Cai et al., 2009). Dysferlin also interacts with annexin A1 and A2, involved in membrane trafficking and actin organization (Lek et al., 2012), Ca_*v*_1.1 L-type calcium channels (Ampong et al., 2005), vinculin, which acts as a link between actin filament and plama membrane (de Morrée et al., 2010), and syntaxin 4, a protein that facilitates the docking and fusion of glucose transporter type 4 (GLUT4) vesicles with the plasma membrane (Evesson et al., 2010; Codding et al., 2016; Drescher et al., 2023).

In the central nervous system, dysferlin has been observed to accumulate in endothelial cells near sclerosis lesions (Hochmeister et al., 2006), as well as within amyloid-beta plaques in people with Alzheimer’s disease (Galvin et al., 2006). In addition, in humans, certain genetic variations of the Dysf gene have been associated with an increased risk of developing Alzheimer’s disease, and Dysf mRNA expression has been shown to be increased in the brains of people with Alzheimer’s disease (Chen et al., 2015). Interestingly, OTOF was identified as a downregulated gene in a human-mouse chimeric model of Alzheimer’s disease using genome-wide expression analysis, suggesting an essential function of otoferlin in the central nervous system (Espuny-Camacho et al., 2017).

Myoferlin (Fer1L3) is also ubiquitously expressed in skeletal and cardiac muscle and in the placenta (Davis et al., 2000). Myoferlin is involved in the fusion of myoblasts, in repair and regeneration, and in muscle cell membrane growth (Doherty et al., 2005). Myoferlin may be involved in the maintenance of transverse tubule function (Demonbreun et al., 2014). Between the C2-C and C2D, a domain consisting of two long β-sheets is called the dysferlin (DysF) domain, and a FerA domain, consisting of α-helices, is located before the FerB domain (Figure 10). The exact role of the DysF domain is unknown but must be significant, as mutations in this domain cause myopathies (Sula et al., 2014). Full information about the ferlin family is available in a recent review by Peulen et al. (2019).

## 7. Otoferlin-disease management and prospects

Auditory Neuropathy Spectrum Disorder (ANSD) is most often suspected after hearing loss is detected at birth, especially if otoacoustic emissions, temporal bone MRI, and CT scans are normal. This deafness may be detected later if the otoacoustic emissions at birth are falsely reassuring. The diagnosis is confirmed by molecular genetic testing, which shows biallelic pathogenic variants in OTOF. Two phenotypes are possible: OTOF-related ANSD, with severe to profound bilateral hearing loss (>70 dB), and temperature-sensitive ANSD (TS ANSD), with normal to moderate hearing loss (0–69 dB) at normal body temperature and worsening to profound hearing loss during hyperthermia. In these patients, prevention of hyperthermia by early use of antipyretics for infection and avoidance of exposure to high temperatures is important (Azaiez et al., 2021).

As we have seen above, in OTOF-related ANSD, the defects are presynaptic at the level of the hair cells. In these situations, the cochlear nerve is intact and functional. This is a prerequisite for cochlear implantation. It is currently the only option for restoring hearing in patients with severe to profound hearing loss. Early implantation is recommended after the diagnosis of OTOF-related auditory neuropathy with severe to profound hearing loss. It involves surgery. A foreign body is implanted under the skin, and an electrode array is placed in the cochlea for life (De Seta et al., 2022). It requires the wearing of an external magnetic processor. The risk of infection or failure requiring re-intervention and the limited autonomy of the device are significant limitations of this therapy. We can also point out the potential limitations of speech recognition in noise. In OTOF-related ANSD, audiometric results are known since 2005 (Loundon et al., 2005). They can be considered of high quality since the thresholds for sound perception in silence from 500 to 2000 Hz vary on average from 25 to 45 dB SPL, depending on the series (Loundon et al., 2005; Rouillon et al., 2006; Zhang et al., 2016; Chen et al., 2018; Zheng and Liu, 2020). Regarding speech recognition, which is the main goal of cochlear implantation, discrimination scores are mostly >90%. There is no evidence that cochlear implantation outcomes are correlated with distinct OTOF genotypes (Zheng and Liu, 2020).

Gene therapy for congenital deafness has been expanding in recent years, with the aim of achieving a therapy with better hearing outcomes without the limitations of cochlear implant. A truncated form of the OTOF gene was shown to rescue hearing and balance in zebrafish (Chatterjee et al., 2015). Since then, several studies have been interested in genetic therapy using recombinant dual AAV vectors (adeno-associated viruses) to encode full-length otoferlin (Akil et al., 2019; Al-Moyed et al., 2019; Rankovic et al., 2020). These studies gave very encouraging results by showing significant restoration of hearing in mice. Recently, recombinant dual AAV vectors encoding for the whole human cochlear otoferlin were used to restore hearing in mice to near wild-type levels for 6 months (Tang et al., 2022). This raises the prospect of treatment by gene therapy in humans for DFNB9. The main limitations are the early age of AAV treatment used in most of these studies, which would correspond to an *in utero* AAV injection in humans, and the weak restoration of the ABR wave-1 amplitude, which could limit speech intelligibility and discrimination, particularly in noise, as the main current defect known for cochlear implants. The weak restoration of wave-1 amplitude during AAV gene therapy is likely due to an uncomplete rescue of the loss of ribbon synapses in the absence of functional otoferlin (Stalmann et al., 2021). Note that a reduction in spiral ganglion neurons with apoptosis is also a secondary defect caused by a lack of otoferlin in IHCs (Tsuzuki et al., 2023). Currently, several biotech companies (SENSORION, NCT05402813 and AKOUOS–NCT05572073) are in the pre-clinical phase for the development of gene therapy for otoferlin mutations in humans.

## 8. Otoferlin and cancer

### 8.1. Ferlins

As we have seen, ferlins are a group of proteins that play an important role in several membrane-related processes that are crucial for cell survival and signaling. These processes include endocytosis, exocytosis, recycling, and membrane repair (Bansal et al., 2003; Roux et al., 2006; Bernatchez et al., 2009; Redpath et al., 2016). The link between these processes and cancer is through their effect on cell Ca^2+^ signaling and development, which allows tumors to form and cancer cells to adapt to a hostile environment. Despite their importance, ferlins have not been extensively studied in the context of cancer. However, recent research has shown that all ferlin genes are modulated in different types of cancer (Peulen et al., 2019). Myoferlin and fer1l4 genes are more commonly upregulated (Barnhouse et al., 2018; You et al., 2020). Myoferlin has been found to be highly expressed in several types of cancer, including kidney, liver, pancreatic, breast, and head and neck squamous cell carcinomas (Wang et al., 2013; Kumar et al., 2016; Blomme et al., 2017; Hermanns et al., 2017; Song et al., 2017).

### 8.2. Myoferlin

The literature on ferlins in cancer only started about 10 years ago, although the proteins have been described for over 20 years. Most of the work has focused on myoferlin, which appears to be the ferlin with the most important role in cancer. The fact that ferlin expression can be either a good prognostic factor (breast cancer) or a poor prognostic factor (kidney, head and neck cancer) is one of the most surprising elements.

In the study of Yadav et al. (2017) which shows that when cells are treated with IL-6, myoferlin dissociates from EHD2 and binds to activated STAT3, a protein involved in cell signaling. The study found that depletion of myoferlin did not affect STAT3 phosphorylation, but blocked its translocation to the nucleus. The study also found that myoferlin knockdown significantly reduced IL-6-mediated tumor cell migration, tumor sphere formation, and the population of cancer stem cells *in vitro*. In addition, myoferlin knockdown significantly reduced IL-6-mediated tumor growth and metastasis. In 211 patients with head and neck squamous cell carcinoma (HNSCC), Kumar et al. (2016) investigated the association of myoferlin with disease progression and patient outcome. The results showed that nuclear myoferlin expression is associated with poor overall survival and an increased risk of death, as well as tumor recurrence, perineural invasion, extracapsular spread, a higher T stage, and distant metastasis. The study also found a direct association between nuclear myoferlin expression and IL-6 and an inverse association with HPV status. Patients with both nuclear myoferlin expression and high levels of IL-6 and those with HPV-negative/myoferlin-positive tumors had the worst survival. These results suggest that nuclear myoferlin expression independently predicts poor clinical outcomes in these patients.

By modeling the effect of myoferlin on tumor cell invasion through altered regulation of metalloproteinase production, Eisenberg et al. (2011) identified a role for myoferlin in promoting invasive behavior in breast cancer cells. The identification of myoferlin as a key regulator of EGFR (Epidermal Growth Factor Receptor) activity through inhibition of non-clathrin endocytosis in breast cancer cells was important in understanding the molecular mechanisms involved in cancer growth (Turtoi et al., 2013). The production of myoferlin-silenced tumor cells has provided another element that may help explain the role of ferlin: these tumors lacked functional blood vessels, an effect that may be due to a reduction in VEGFA exocytosis (Fahmy et al., 2016). This may explain its strong association with cancer prognosis, as an independent prognosis factor in kidney (Song et al., 2017).

### 8.3. Otoferlin

**FIGURE 11:**
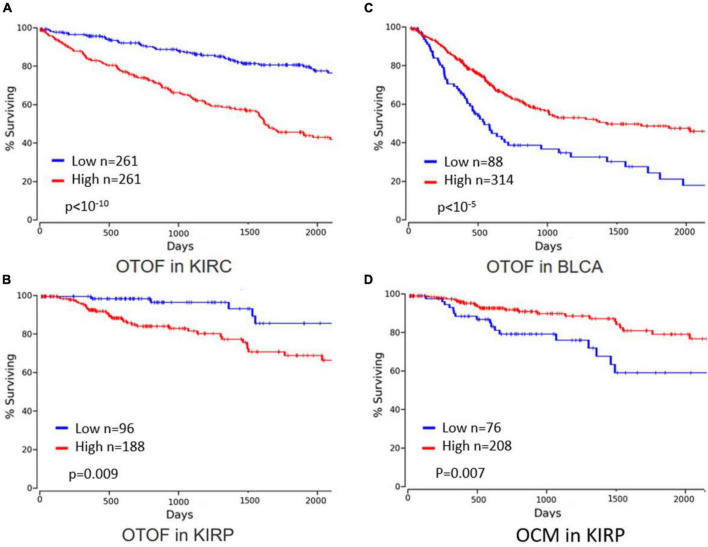
Survival data linked to otoferlin and oncomodulin expression levels (graphs were built after bioinformatics analysis using OncoLnc, 2023). Year overall survival was better for patients with lower otoferlin gene expression in **(A)** kidney renal clear cell carcinoma (KIRC) and **(B)** kidney renal papillary cell carcinoma (KIRP), whereas survival was better for patients with higher otoferlin gene expression in **(C)** bladder urothelial carcinoma (BLCA). High levels of oncomoduline (OCM), a parvalbumin-family calcium-binding protein highly expressed in hair cells, in KIRP **(D)** is associated with better 5-year overall survival.

Currently, there is limited information available on otoferlin. Involvement of IL-6 in inner ear damage during noise trauma or cisplatin treatment has been reported (So et al., 2007; Wakabayashi et al., 2010), but a possible link with otoferlin function during disease progression has not yet been explored. Using OncoLnc, a tool for exploring correlations between the expression of mRNAs, mRNAs, and IncRNAs, we analyzed 5-year overall survival in 21 cancer types (Anaya, 2016). In three cancers, we found a significant impact of otoferlin expression on survival: in renal clear cell, papillary cell carcinoma, and in bladder urothelial carcinoma (Figure 11). Interestingly, otoferlin expression was either a protective or a risk factor depending on the histology of the cancer. Given the differences in 5-year survival, it seems appropriate to consider using otoferlin expression as a prognostic biomarker to guide therapeutic decisions.

In clear cell renal cell carcinoma, Cox et al. (2021) showed in a study of 79 patients that otoferlin was an indicator of tumor staging and a prognostic biomarker for cancer-specific survival. In 25 patients with oral squamous cell carcinoma, Kraus showed complete suppression of otoferlin expression in malignant tissue (Kraus et al., 2022). Taken together, these results suggest a role for otoferlin in the carcinogenesis of these tumors, which deserves investigation to confirm and understand its exact role, especially as it appears to vary according to tumor site. Studies are needed to ensure that otoferlin is an independent prognostic factor for overall survival. This could lead to new cancer therapies targeting this protein, which are already underway for myoferlin (Zhang et al., 2018; Li et al., 2019; He et al., 2021).

### 8.4. Oncomodulin

Hair cells, notably OHCs, highly express another protein, which has also been used as a prognostic marker in cancer: oncomodulin (OCM). OCM is a small EF-hand Ca^2+^-binding protein (CaBP) of approximately 12 kDa. It belongs to the parvalbumin family. It is the β-isoform of parvalbumin. It shares at least 53% sequence identity with alpha-parvalbumin (PVALB) (Climer et al., 2019). OCM has an unusually restrictive post-embryonic expression pattern in mammals, mostly restricted to subsets of sensory hair cells in the inner ear and more recently found in certain subtypes of immune cells. Oncomodulin is essential for normal hearing function since its deletion in mice leads to progressive deafness due to alterations in the mechanical amplification function of OHCs (Tong et al., 2016). We recall that OHC lateral mechanical stiffness and motility have long been recognized as regulated by calcium acting on the actin-based cortical cytoskeleton (Dulon et al., 1990). Oncomodulin, acting as a CaBP, may be essential in sculpting calcium signals important for F-actin remodeling and membrane mechanics in OHCs, a role somewhat similar to that of otoferlin in IHCs, as proposed in our review.

The initial discovery of OCM as an oncoprotein in cancer tissue and its similarity to calmodulin as a CaBP led to the term “oncomodulin” (Durkin et al., 1983). OCM was considered oncogenic because no expression was detected in normal post-embryonic tissue. However, after its initial discovery, OCM was identified as a major protein in sensory cells of the cochlea. Studies have suggested that OCM may play a role in cell proliferation, and recently, Yin et al. (2006) identified oncomodulin as a potent macrophage-derived growth factor for retinal ganglion cells (RGCs) and other neurons. Oncomodulin also stimulates the outgrowth of peripheral sensory neurons.

## 9. Discussion-conclusion

Otoferlin, a protein mainly produced in the brain and inner ear, plays a key role in hearing. More than 220 mutations causing DFNB9-type deafness have been identified so far. Many of these mutations alter the folding, stability, or function of the protein, resulting in severe to profound hearing loss associated with a defect in synaptic transmission between the IHC and the nerve fibers of the auditory nerve.

An intriguing question is: why have auditory and vestibular hair cells selected otoferlin as a key Ca^2+^-sensor to control exocytosis of synaptic vesicles instead of the classical synaptotagmins found in central synapses? The need to encode tiny graded microphonic potentials spanning over a large range of amplitude (several dozens of dB) by graded multivesicular release at each ribbon synapses and the requirement to sustain an extremely high rate of vesicle release by an efficient vesicular replenishment are probably the two main factors explaining the use of a large multi-C2 Ca^2+^ sensing protein such as otoferlin. Another important property of otoferlin is its capability to function as a high affinity large-range Ca^2+^ sensor as compared to synaptotagmins. In our review, we propose otoferlin as a Ca^2+^-dependent mechanical interactor between the membranous F-actin network and the synaptic vesicles at the hair cell ribbon. This protein would confer Ca^2+^-dependent oscillatory movement of the ribbon, whose resonant frequency would depend on their size (mass), tuning the firing frequency of the postsynaptic fibers.

In this context, it is important to note that otoferlin belongs to the ferlin protein family in humans: dysferlin, myoferlin, fer1L4, fer1L5, and fer1L6, all of which being expressed in mechanically active or contracting cells. These proteins share common features such as the presence of multiple C2 domains, FerA domains, and membrane anchoring through their C-terminal domain, and are thought to play a role in calcium-triggered membrane fusion and trafficking. Although otoferlin is expressed in the central nervous system, in particular in the cortex and amygdala, its role in brain tissues remains unknown. Interestingly, a genome-wide expression analysis of a human-mouse chimeric model of Alzheimer’s disease identified OTOF as a downregulated gene, suggesting a critical function of otoferlin in the central nervous system. Dysferlin has been observed to accumulate in endothelial cells adjacent to sclerotic lesions and within amyloid-beta plaques in individuals with Alzheimer’s disease. These findings highlight the need for further research into the role of ferlin proteins in the central nervous system, particularly their potential involvement in neurodegenerative diseases such as Alzheimer’s disease.

Also, otoferlin, like many of the other ferlins, plays a crucial role in membrane-related processes that are important for cell survival and signaling. Studies have suggested a link between ferlins and cancer through their effect on cell signaling and development, allowing tumors to form and cancer cells to adapt to a hostile environment. Despite their importance, ferlins have not been extensively studied in the context of cancer. Data show that otoferlin expression is significantly associated with survival in specific cancer types, including clear cell and papillary cell renal carcinoma, and urothelial bladder cancer. These results suggest a role for otoferlin in the carcinogenesis of these tumors, which needs further investigation to confirm and understand its exact role, especially as it appears to vary according to tumor site. This could lead to new cancer therapies targeting this protein.

## Author contributions

J-CL and DD wrote the manuscript. Both authors contributed to the article and approved the submitted version.

## References

[B1] Achanzar W. E. Ward S. (1997). A nematode gene required for sperm vesicle fusion. *J. Cell Sci.* 110(Pt 9), 1073–1081. 10.1242/jcs.110.9.1073 9175703

[B2] Ajmal A. Humayun M. H. Hassan M. U. Anwar M. S. (2023). *Properties of rubber balloons: addtitional notes.* Available online at: https://physlab.org/wp-content/uploads/2023/01/student_manual_additional_notes.pdf (accessed March 29, 2023).

[B3] Akil O. (2020). Dual and triple AAV delivery of large therapeutic gene sequences into the inner ear. *Hear. Res.* 394:107912. 10.1016/j.heares.2020.107912 32067799

[B4] Akil O. Dyka F. Calvet C. Emptoz A. Lahlou G. Nouaille S. (2019). Dual AAV-mediated gene therapy restores hearing in a DFNB9 mouse model. *Proc. Natl. Acad. Sci. U.S.A.* 116 4496–4501. 10.1073/pnas.1817537116 30782832 PMC6410774

[B5] Al-Moyed H. Cepeda A. P. Jung S. Moser T. Kügler S. Reisinger E. (2019). A dual-AAV approach restores fast exocytosis and partially rescues auditory function in deaf otoferlin knock-out mice. *EMBO Mol. Med.* 11:e9396. 10.15252/emmm.201809396 30509897 PMC6328916

[B6] Ampong B. N. Imamura M. Matsumiya T. Yoshida M. Takeda S. (2005). Intracellular localization of dysferlin and its association with the dihydropyridine receptor. *Acta Myol.* 24 134–144. 16550931

[B7] Anaya J. (2016). OncoLnc: linking TCGA survival data to mRNAs, miRNAs, and lncRNAs. *Peer J. Comput. Sci.* 2:e67. 10.7717/peerj-cs.67

[B8] Anderson L. V. Davison K. Moss J. A. Young C. Cullen M. J. Walsh J. (1999). Dysferlin is a plasma membrane protein and is expressed early in human development. *Hum. Mol. Genet* 8 855–861. 10.1093/hmg/8.5.855 10196375

[B9] Azaiez H. Thorpe R. K. Smith R. J. (2021). *OTOF-related deafness.* Seattle, WA: University of Washington. 20301429

[B10] Bacon S. (1990). Effect of masker level on overshoot. *J. Acoust. Soc. Am.* 88 698–702. 10.1121/1.399773 2212293

[B11] Bacon S. Moore B. (1987). Transient masking and the temporal course of simultaneous tone-on-tone masking. *J. Acoust. Soc. Am.* 81 1073–1077. 10.1121/1.395125 3571723

[B12] Baig S. M. Koschak A. Lieb A. Gebhart M. Dafinger C. Nürnberg G. (2011). Loss of Ca(v)1.3 (CACNA1D) function in a human channelopathy with bradycardia and congenital deafness. *Nat. Neurosci.* 14 77–84. 10.1038/nn.2694 21131953

[B13] Bansal D. Miyake K. Vogel S. S. Groh S. Chen C.-C. Williamson R. (2003). Defective membrane repair in dysferlin-deficient muscular dystrophy. *Nature* 423 168–172. 10.1038/nature01573 12736685

[B14] Barnhouse V. R. Weist J. L. Shukla V. C. Ghadiali S. N. Kniss D. A. Leight J. L. (2018). Myoferlin regulates epithelial cancer cell plasticity and migration through autocrine TGF-β1 signaling. *Oncotarget* 9 19209–19222. 10.18632/oncotarget.24971 29721195 PMC5922389

[B15] Barthélémy F. Defour A. Lévy N. Krahn M. Bartoli M. (2018). Muscle cells fix breaches by orchestrating a membrane repair ballet. *J. Neuromuscul. Dis.* 5 21–28. 10.3233/JND-170251 29480214 PMC5836414

[B16] Bashir R. Britton S. Strachan T. Keers S. Vafiadaki E. Lako M. (1998). A gene related to *Caenorhabditis elegans* spermatogenesis factor fer-1 is mutated in limb-girdle muscular dystrophy type 2B. *Nat. Genet.* 20 37–42. 10.1038/1689 9731527

[B17] Becker L. Schnee M. E. Niwa M. Sun W. Maxeiner S. Talaei S. (2018). The presynaptic ribbon maintains vesicle populations at the hair cell afferent fiber synapse. *eLife* 7:e30241. 10.7554/eLife.30241 29328021 PMC5794257

[B18] Bernatchez P. N. Sharma A. Kodaman P. Sessa W. C. (2009). Myoferlin is critical for endocytosis in endothelial cells. *Am. J. Physiol. Cell Physiol.* 297 C484–C492. 10.1152/ajpcell.00498.2008 19494235 PMC2740391

[B19] Beurg M. Michalski N. Safieddine S. Bouleau Y. Schneggenburger R. Chapman E. R. (2010). Control of exocytosis by synaptotagmins and otoferlin in auditory hair cells. *J. Neurosci.* 30 13281–13290. 10.1523/JNEUROSCI.2528-10.2010 20926654 PMC3088501

[B20] Beurg M. Safieddine S. Roux I. Bouleau Y. Petit C. Dulon D. (2008). Calcium- and otoferlin-dependent exocytosis by immature outer hair cells. *J. Neurosci.* 28 1798–1803. 10.1523/JNEUROSCI.4653-07.2008 18287496 PMC6671446

[B21] Blomme A. Costanza B. de Tullio P. Thiry M. Van Simaeys G. Boutry S. (2017). Myoferlin regulates cellular lipid metabolism and promotes metastases in triple-negative breast cancer. *Oncogene* 36 2116–2130. 10.1038/onc.2016.369 27775075

[B22] Bornschein G. Schmidt H. (2018). Synaptotagmin Ca^2+^ sensors and their spatial coupling to presynaptic Cav channels in central cortical synapses. *Front. Mol. Neurosci.* 11:494. 10.3389/fnmol.2018.00494 30697148 PMC6341215

[B23] Cai C. Weisleder N. Ko J.-K. Komazaki S. Sunada Y. Nishi M. (2009). Membrane repair defects in muscular dystrophy are linked to altered interaction between MG53, caveolin-3, and dysferlin. *J. Biol. Chem.* 284 15894–15902. 10.1074/jbc.M109.009589 19380584 PMC2708885

[B24] Calvet C. Peineau T. Benamer N. Cornille M. Lelli A. Plion B. (2022). The SNARE protein SNAP-25 is required for normal exocytosis at auditory hair cell ribbon synapses. *iScience* 25:105628. 10.1016/j.isci.2022.105628 36483015 PMC9722480

[B25] Chapman E. R. (2008). How does synaptotagmin trigger neurotransmitter release? *Annu. Rev. Biochem.* 77 615–641. 18275379 10.1146/annurev.biochem.77.062005.101135

[B26] Chatterjee M. Smith R. (1993). Physiological overshoot and the compound action potential. *Hear. Res.* 69 45–54. 10.1016/0378-5955(93)90092-f 8226349

[B27] Chatterjee P. Padmanarayana M. Abdullah N. Holman C. L. LaDu J. Tanguay R. L. (2015). Otoferlin deficiency in zebrafish results in defects in balance and hearing: rescue of the balance and hearing phenotype with full-length and truncated forms of mouse otoferlin. *Mol. Cell Biol.* 35 1043–1054. 10.1128/MCB.01439-14 25582200 PMC4333087

[B28] Chen J. A. Wang Q. Davis-Turak J. Li Y. Karydas A. M. Hsu S. C. (2015). A multiancestral genome-wide exome array study of Alzheimer disease, frontotemporal dementia, and progressive supranuclear palsy. *JAMA Neurol.* 72 414–422. 10.1001/jamaneurol.2014.4040 25706306 PMC4397175

[B29] Chen K. Liu M. Wu X. Zong L. Jiang H. (2018). Targeted next generation sequencing reveals OTOF mutations in auditory neuropathy spectrum disorder. *Int. J. PediatrOtorhinolaryngol.* 115 19–23. 10.1016/j.ijporl.2018.09.008 30368385

[B30] Choi B. Y. Ahmed Z. M. Riazuddin S. Bhinder M. A. Shahzad M. Husnain T. (2009). Identities and frequencies of mutations of the otoferlin gene (OTOF) causing DFNB9 deafness in Pakistan. *Clin. Genet.* 75 237–243. 10.1111/j.1399-0004.2008.01128.x 19250381 PMC3461579

[B31] Climer L. K. Cox A. M. Reynolds T. J. Simmons D. D. (2019). Oncomodulin: the enigmatic Parvalbumin protein. *Front. Mol. Neurosci.* 12:235. 10.3389/fnmol.2019.00235 31649505 PMC6794386

[B32] Codding S. J. Marty N. Abdullah N. Johnson C. P. (2016). Dysferlin binds SNAREs (Soluble N-Ethylmaleimide-sensitive Factor (NSF) Attachment Protein Receptors) and stimulates membrane fusion in a calcium-sensitive manner. *J. Biol. Chem.* 291 14575–14584. 10.1074/jbc.M116.727016 27226605 PMC4938179

[B33] Corbalan-Garcia S. Gómez-Fernández J. C. (2014). Signaling through C2 domains: more than one lipid target. *Biochim. Biophys. Acta* 1838 1536–1547. 10.1016/j.bbamem.2014.01.008 24440424

[B34] Courtney K. Vevea J. Li Y. Wu Z. Zhang Z. Chapman E. (2021). Synaptotagmin 1 oligomerization via the juxtamembrane linker regulates spontaneous and evoked neurotransmitter release. *Proc. Natl. Acad. Sci. U.S.A.* 118:e2113859118. 10.1073/pnas.2113859118 34810248 PMC8694047

[B35] Cox A. Tolkach Y. Stein J. Kristiansen G. Ritter M. Ellinger J. (2021). Otoferlin is a prognostic biomarker in patients with clear cell renal cell carcinoma: a systematic expression analysis. *Int. J. Urol.* 28 424–431. 10.1111/iju.14486 33465825

[B36] Davis D. B. Delmonte A. J. Ly C. T. McNally E. M. (2000). Myoferlin, a candidate gene and potential modifier of muscular dystrophy. *Hum. Mol. Genet.* 9 217–226. 10.1093/hmg/9.2.217 10607832

[B37] de Morrée A. Hensbergen P. J. van Haagen H. H. H. B. M. Dragan I. Deelder A. M.’t Hoen P. A. C. (2010). Proteomic analysis of the dysferlin protein complex unveils its importance for sarcolemmal maintenance and integrity. *PLoS One* 5:e13854. 10.1371/journal.pone.0013854 21079765 PMC2974636

[B38] De Seta D. Daoudi H. Torres R. Ferrary E. Sterkers O. Nguyen Y. (2022). Robotics, automation, active electrode arrays, and new devices for cochlear implantation: a contemporary review. *Hear. Res.* 414:108425. 10.1016/j.heares.2021.108425 34979455

[B39] Demonbreun A. R. Rossi A. E. Alvarez M. G. Swanson K. E. Deveaux H. K. Earley J. U. (2014). Dysferlin and Myoferlin regulate transverse tubule formation and glycerol sensitivity. *Am. J. Pathol.* 184 248–259. 10.1016/j.ajpath.2013.09.009 24177035 PMC3873498

[B40] Doherty K. R. Cave A. Davis D. B. Delmonte A. J. Posey A. Earley J. U. (2005). Normal myoblast fusion requires myoferlin. *Development* 132 5565–5575. 10.1242/dev.02155 16280346 PMC4066872

[B41] Drescher D. G. Drescher M. J. Selvakumar D. Annam N. P. (2023). Analysis of dysferlin direct interactions with putative repair proteins links apoptotic signaling to Ca^2+^ elevation via PDCD6 and FKBP8. *Int. J. Mol. Sci.* 24:4707. 10.3390/ijms24054707 36902136 PMC10002499

[B42] Dulon D. Papal S. Patni P. Cortese M. Vincent P. F. Tertrais M. (2018). Clarin-1 gene transfer rescues auditory synaptopathy in model of Usher syndrome. *J. Clin. Invest.* 128 3382–3401. 10.1172/JCI94351 29985171 PMC6063508

[B43] Dulon D. Safieddine S. Jones S. M. Petit C. (2009). Otoferlin is critical for a highly sensitive and linear calcium-dependent exocytosis at vestibular hair cell ribbon synapses. *J. Neurosci.* 29 10474–10487. 10.1523/JNEUROSCI.1009-09.2009 19710301 PMC2966717

[B44] Dulon D. Zajic G. Schacht J. (1990). Increasing intracellular free calcium induces circumferential contractions in isolated cochlear outer hair cells. *J. Neurosci.* 10 1388–1397. 10.1523/JNEUROSCI.10-04-01388.1990 2109787 PMC6570211

[B45] Duncker S. V. Franz C. Kuhn S. Schulte U. Campanelli D. Brandt N. (2013). Otoferlin couples to clathrin-mediated endocytosis in mature cochlear inner hair cells. *J. Neurosci.* 33 9508–9519. 10.1523/JNEUROSCI.5689-12.2013 23719817 PMC3676539

[B46] Durkin J. P. Brewer L. M. MacManus J. P. (1983). Occurrence of the tumor-specific, calcium-binding protein, oncomodulin, in virally transformed normal rat kidney cells. *Cancer Res.* 43 5390–5394. 6311406

[B47] Eisenberg M. C. Kim Y. Li R. Ackerman W. E. Kniss D. A. Friedman A. (2011). Mechanistic modeling of the effects of myoferlin on tumor cell invasion. *Proc. Natl. Acad. Sci. U.S.A.* 108 20078–20083. 10.1073/pnas.1116327108 22135466 PMC3250187

[B48] Espuny-Camacho I. Arranz A. M. Fiers M. Snellinx A. Ando K. Munck S. (2017). Hallmarks of Alzheimer’s disease in stem-cell-derived human neurons transplanted into mouse brain. *Neuron* 93 1066.e8–1081.e8. 10.1016/j.neuron.2017.02.001 28238547

[B49] Evesson F. J. Peat R. A. Lek A. Brilot F. Lo H. P. Dale R. C. (2010). Reduced plasma membrane expression of dysferlin mutants is attributed to accelerated endocytosis via a syntaxin-4-associated pathway. *J. Biol. Chem.* 285 28529–28539. 10.1074/jbc.M110.111120 20595382 PMC2937879

[B50] Fahmy K. Gonzalez A. Arafa M. Peixoto P. Bellahcène A. Turtoi A. (2016). Myoferlin plays a key role in VEGFA secretion and impacts tumor-associated angiogenesis in human pancreas cancer. *Int. J. Cancer* 138 652–663. 10.1002/ijc.29820 26311411

[B51] Galvin J. E. Palamand D. Strider J. Milone M. Pestronk A. (2006). The muscle protein dysferlin accumulates in the Alzheimer brain. *Acta Neuropathol.* 112 665–671. 10.1007/s00401-006-0147-8 17024495 PMC1705477

[B52] Graydon C. W. Manor U. Kindt K. S. (2017). *In vivo* ribbon mobility and turnover of ribeye at zebrafish hair cell synapses. *Sci. Rep.* 7:7467. 10.1038/s41598-017-07940-z 28785118 PMC5547071

[B53] Gregory F. D. Bryan K. E. Pangršič T. Calin-Jageman I. E. Moser T. Lee A. (2011). Harmonin inhibits presynaptic Cav1.3 Ca^2+^ channels in mouse inner hair cells. *Nat. Neurosci.* 14 1109–1111. 10.1038/nn.2895 21822269 PMC3164920

[B54] Haddadzade N. H. Pourbakht A. Rahbar N. Haghani H. (2021). Brainstem representation of auditory overshoot in guinea pigs using auditory brainstem responses. *Iran J. Child Neurol.* 15 41–56. 10.22037/ijcn.v15i2.26241 36213160 PMC9376021

[B55] Hams N. Padmanarayana M. Qiu W. Johnson C. P. (2017). Otoferlin is a multivalent calcium-sensitive scaffold linking SNAREs and calcium channels. *Proc. Natl. Acad. Sci. U.S.A.* 114 8023–8028. 10.1073/pnas.1703240114 28696301 PMC5544299

[B56] Harsini F. M. Chebrolu S. Fuson K. L. White M. A. Rice A. M. Sutton R. B. (2018). FerA is a membrane-associating four-helix bundle domain in the ferlin family of membrane-fusion proteins. *Sci. Rep.* 8:10949. 10.1038/s41598-018-29184-1 30026467 PMC6053371

[B57] He Y. Kan W. Li Y. Hao Y. Huang A. Gu H. (2021). A potent and selective small molecule inhibitor of myoferlin attenuates colorectal cancer progression. *Clin. Transl. Med.* 11:e289. 10.1002/ctm2.289 33634965 PMC7868085

[B58] Heidrych P. Zimmermann U. Bress A. Pusch C. M. Ruth P. Pfister M. (2008). Rab8b GTPase, a protein transport regulator, is an interacting partner of otoferlin, defective in a human autosomal recessive deafness form. *Hum. Mol. Genet.* 17 3814–3821. 10.1093/hmg/ddn279 18772196

[B59] Heidrych P. Zimmermann U. Kuhn S. Franz C. Engel J. Duncker S. V. (2009). Otoferlin interacts with myosin VI: Implications for maintenance of the basolateral synaptic structure of the inner hair cell. *Hum. Mol. Genet.* 18 2779–2790. 10.1093/hmg/ddp213 19417007

[B60] Helfmann S. Neumann P. Tittmann K. Moser T. Ficner R. Reisinger E. (2011). The crystal structure of the C_2_A domain of otoferlin reveals an unconventional top loop region. *J. Mol. Biol.* 406 479–490. 10.1016/j.jmb.2010.12.031 21216247

[B61] Hermanns C. Hampl V. Holzer K. Aigner A. Penkava J. Frank N. (2017). The novel MKL target gene myoferlin modulates expansion and senescence of hepatocellular carcinoma. *Oncogene* 36 3464–3476. 10.1038/onc.2016.496 28114277

[B62] Hernández-Deviez D. J. Howes M. T. Laval S. H. Bushby K. Hancock J. F. Parton R. G. (2008). Caveolin regulates endocytosis of the muscle repair protein, dysferlin. *J. Biol. Chem.* 283 6476–6488. 10.1074/jbc.M708776200 18096699

[B63] Highstein S. M. Holstein G. R. Mann M. A. Rabbitt R. D. (2014). Evidence that protons act as neurotransmitters at vestibular hair cell-calyx afferent synapses. *Proc. Natl. Acad. Sci. U.S.A.* 111 5421–5426. 10.1073/pnas.1319561111 24706862 PMC3986198

[B64] Hochmeister S. Grundtner R. Bauer J. Engelhardt B. Lyck R. Gordon G. (2006). Dysferlin is a new marker for leaky brain blood vessels in multiple sclerosis. *J. Neuropathol. Exp. Neurol.* 65 855–865. 10.1097/01.jnen.0000235119.52311.16 16957579

[B65] Hofhuis J. Bersch K. Büssenschütt R. Drzymalski M. Liebetanz D. Nikolaev V. O. (2017). Dysferlin mediates membrane tubulation and links T-tubule biogenesis to muscular dystrophy. *J. Cell Sci.* 130 841–852. 10.1242/jcs.198861 28104817

[B66] Holt J. C. Chatlani S. Lysakowski A. Goldberg J. M. (2007). Quantal and nonquantal transmission in calyx-bearing fibers of the turtle posterior crista. *J. Neurophysiol.* 98 1083–1101. 10.1152/jn.00332.2007 17596419 PMC3397384

[B67] Hudspeth A. J. Issa N. P. (1996). Confocal-microscopic visualization of membrane addition during synaptic exocytosis at presynaptic active zones of hair cells. *Cold Spring Harb. Symp. Quant. Biol.* 61 303–307. 9246459

[B68] Iwasa Y.-I. Nishio S.-Y. Sugaya A. Kataoka Y. Kanda Y. Taniguchi M. (2019). OTOF mutation analysis with massively parallel DNA sequencing in 2,265 Japanese sensorineural hearing loss patients. *PLoS One* 14:e0215932. 10.1371/journal.pone.0215932 31095577 PMC6522017

[B69] Jean P. Lopez de la Morena D. Michanski S. Jaime Tobón L. M. Chakrabarti R. Picher M. M. (2018). The synaptic ribbon is critical for sound encoding at high rates and with temporal precision. *eLife* 7:e29275. 10.7554/eLife.29275 29328020 PMC5794258

[B70] Jiménez J. L. Bashir R. (2007). In silico functional and structural characterisation of ferlin proteins by mapping disease-causing mutations and evolutionary information onto three-dimensional models of their C2 domains. *J. Neurol. Sci.* 260 114–123. 10.1016/j.jns.2007.04.016 17512949

[B71] Johnson C. P. Chapman E. R. (2010). Otoferlin is a calcium sensor that directly regulates SNARE-mediated membrane fusion. *J. Cell Biol.* 191 187–197. 10.1083/jcb.201002089 20921140 PMC2953437

[B72] Jumper J. Evans R. Pritzel A. Green T. Figurnov M. Ronneberger O. (2021). Highly accurate protein structure prediction with AlphaFold. *Nature* 596 583–589. 10.1038/s41586-021-03819-2 34265844 PMC8371605

[B73] Jung S. Maritzen T. Wichmann C. Jing Z. Neef A. Revelo N. H. (2015). Disruption of adaptor protein 2μ (AP-2μ) in cochlear hair cells impairs vesicle reloading of synaptic release sites and hearing. *EMBO J.* 34 2686–2702. 10.15252/embj.201591885 26446278 PMC4641533

[B74] Kim B. J. Jang J. H. Han J. H. Park H.-R. Oh D. Y. Lee S. (2018). Mutational and phenotypic spectrum of OTOF-related auditory neuropathy in Koreans: eliciting reciprocal interaction between bench and clinics. *J. Transl. Med.* 16:330. 10.1186/s12967-018-1708-z 30482216 PMC6260760

[B75] Krahn M. Wein N. Bartoli M. Lostal W. Courrier S. Bourg-Alibert N. (2010). A naturally occurring human minidysferlin protein repairs sarcolemmal lesions in a mouse model of dysferlinopathy. *Sci. Transl. Med.* 2:50ra69. 10.1126/scitranslmed.3000951 20861509

[B76] Kraus D. Weider S. Probstmeier R. Winter J. (2022). Neoexpression of JUNO in oral tumors is accompanied with the complete suppression of four other genes and suggests the application of new biomarker tools. *J. Pers Med.* 12:494. 10.3390/jpm12030494 35330493 PMC8954609

[B77] Kroll J. Jaime Tobón L. M. Vogl C. Neef J. Kondratiuk I. König M. (2019). Endophilin-A regulates presynaptic Ca^2 +^ influx and synaptic vesicle recycling in auditory hair cells. *EMBO J.* 38:e100116. 10.15252/embj.2018100116 30733243 PMC6396150

[B78] Kumar B. Brown N. V. Swanson B. J. Schmitt A. C. Old M. Ozer E. (2016). High expression of myoferlin is associated with poor outcome in oropharyngeal squamous cell carcinoma patients and is inversely associated with HPV-status. *Oncotarget* 7 18665–18677. 10.18632/oncotarget.7625 26919244 PMC4951318

[B79] Lek A. Evesson F. J. Lemckert F. A. Redpath G. M. I. Lueders A.-K. Turnbull L. (2013). Calpains, cleaved mini-dysferlinC72, and L-type channels underpin calcium-dependent muscle membrane repair. *J. Neurosci.* 33 5085–5094. 10.1523/JNEUROSCI.3560-12.2013 23516275 PMC6704986

[B80] Lek A. Evesson F. J. Sutton R. B. North K. N. Cooper S. T. (2012). Ferlins: regulators of vesicle fusion for auditory neurotransmission, receptor trafficking and membrane repair. *Traffic* 13 185–194. 10.1111/j.1600-0854.2011.01267.x 21838746

[B81] Lek A. Lek M. North K. N. Cooper S. T. (2010). Phylogenetic analysis of ferlin genes reveals ancient eukaryotic origins. *BMC Evol. Biol.* 10:231. 10.1186/1471-2148-10-231 20667140 PMC2923515

[B82] Levic S. Bouleau Y. Dulon D. (2011). Developmental acquisition of a rapid calcium-regulated vesicle supply allows sustained high rates of exocytosis in auditory hair cells. *PLoS One* 6:e25714. 10.1371/journal.pone.0025714 21998683 PMC3188563

[B83] Li Y. He Y. Shao T. Pei H. Guo W. Mi D. (2019). Modification and biological evaluation of a series of 1,5-Diaryl-1,2,4-triazole compounds as novel agents against pancreatic cancer metastasis through targeting Myoferlin. *J. Med. Chem.* 62 4949–4966. 10.1021/acs.jmedchem.9b00059 31026162

[B84] Liberman L. D. Wang H. Liberman M. C. (2011). Opposing gradients of ribbon size and AMPA receptor expression underlie sensitivity differences among cochlear-nerve/hair-cell synapses. *J. Neurosci.* 31 801–808. 10.1523/JNEUROSCI.3389-10.2011 21248103 PMC3290333

[B85] Liberman M. C. (1982). Single-neuron labeling in the cat auditory nerve. *Science* 216 1239–1241. 10.1126/science.7079757 7079757

[B86] Liu H. Liu H. Wang L. Song L. Jiang G. Lu Q. (2023). Cochlear transcript diversity and its role in auditory functions implied by an otoferlin short isoform. *Nat. Commun.* 14:3085. 10.1038/s41467-023-38621-3 PMC10227054 37248244

[B87] Liu J. Aoki M. Illa I. Wu C. Fardeau M. Angelini C. (1998). Dysferlin, a novel skeletal muscle gene, is mutated in Miyoshi myopathy and limb girdle muscular dystrophy. *Nat. Genet.* 20 31–36. 10.1038/1682 9731526

[B88] Loundon N. Marcolla A. Roux I. Rouillon I. Denoyelle F. Feldmann D. (2005). Auditory neuropathy or endocochlear hearing loss? *Otol. Neurotol.* 26 748–754. 10.1097/01.mao.0000169044.63970.4a 16015179

[B89] Manchanda A. Bonventre J. A. Bugel S. M. Chatterjee P. Tanguay R. Johnson C. P. (2021). Truncation of the otoferlin transmembrane domain alters the development of hair cells and reduces membrane docking. *Mol. Biol. Cell* 32 1293–1305. 10.1091/mbc.E20-10-0657 33979209 PMC8351550

[B90] Manchanda A. Chatterjee P. Bonventre J. A. Haggard D. E. Kindt K. S. Tanguay R. L. (2019). Otoferlin depletion results in abnormal synaptic ribbons and altered intracellular calcium levels in Zebrafish. *Sci. Rep.* 9:14273. 10.1038/s41598-019-50710-2 31582816 PMC6776657

[B91] Marlin S. Feldmann D. Nguyen Y. Rouillon I. Loundon N. Jonard L. (2010). Temperature-sensitive auditory neuropathy associated with an otoferlin mutation: deafening fever! *Biochem. Biophys. Res. Commun.* 394 737–742. 10.1016/j.bbrc.2010.03.062 20230791

[B92] Matsuda C. Hayashi Y. K. Ogawa M. Aoki M. Murayama K. Nishino I. (2001). The sarcolemmal proteins dysferlin and caveolin-3 interact in skeletal muscle. *Hum. Mol. Genet.* 10 1761–1766. 10.1093/hmg/10.17.1761 11532985

[B93] Matsunaga T. Mutai H. Kunishima S. Namba K. Morimoto N. Shinjo Y. (2012). A prevalent founder mutation and genotype–phenotype correlations of OTOF in Japanese patients with auditory neuropathy. *Clin. Genet.* 82 425–432. 10.1111/j.1399-0004.2012.01897.x 22575033

[B94] McNeil P. L. Kirchhausen T. (2005). An emergency response team for membrane repair. *Nat. Rev. Mol. Cell Biol.* 6 499–505. 10.1038/nrm1665 15928713

[B95] Meese S. Cepeda A. P. Gahlen F. Adams C. M. Ficner R. Ricci A. J. (2017). Activity-dependent phosphorylation by CaMKIIδ alters the Ca^2 +^ affinity of the multi-C2-domain protein otoferlin. *Front. Synaptic Neurosci.* 9:13. 10.3389/fnsyn.2017.00013 29046633 PMC5632675

[B96] Michalski N. Goutman J. D. Auclair S. M. Boutet de Monvel J. Tertrais M. Emptoz A. (2017). Otoferlin acts as a Ca^2+^ sensor for vesicle fusion and vesicle pool replenishment at auditory hair cell ribbon synapses. *eLife* 6:e31013. 10.7554/eLife.31013 29111973 PMC5700815

[B97] Moser T. Starr A. (2016). Auditory neuropathy–neural and synaptic mechanisms. *Nat. Rev. Neurol.* 12 135–149. 10.1038/nrneurol.2016.10 26891769

[B98] Müller I. Struchtrup H. (2002). Inflating a rubber balloon. *Math. Mech. Solids* 7 569–577. 10.1177/108128650200700506

[B99] Nouvian R. Neef J. Bulankina A. V. Reisinger E. Pangršič T. Frank T. (2011). Exocytosis at the hair cell ribbon synapse apparently operates without neuronal SNARE proteins. *Nat. Neurosci.* 14 411–413. 10.1038/nn.2774 21378973

[B100] Omasits U. Ahrens C. H. Müller S. Wollscheid B. (2014). Protter: interactive protein feature visualization and integration with experimental proteomic data. *Bioinformatics* 30 884–886. 10.1093/bioinformatics/btt607 24162465

[B101] OncoLnc, (2023). *OncoLnc.* Available online at: http://www.oncolnc.org/ (accessed on February 1, 2023).

[B102] Özçete ÖD. Moser T. (2021). A sensory cell diversifies its output by varying Ca^2+^ influx-release coupling among active zones. *EMBO J.* 40:e106010. 10.15252/embj.2020106010 33346936 PMC7917556

[B103] Padmanarayana M. Hams N. Speight L. C. Petersson E. J. Mehl R. A. Johnson C. P. (2014). Characterization of the lipid binding properties of Otoferlin reveals specific interactions between PI(4,5)P2 and the C2C and C2F domains. *Biochemistry* 53 5023–5033. 10.1021/bi5004469 24999532 PMC4144714

[B104] Pangrsic T. Lasarow L. Reuter K. Takago H. Schwander M. Riedel D. (2010). Hearing requires otoferlin-dependent efficient replenishment of synaptic vesicles in hair cells. *Nat. Neurosci.* 13 869–876. 10.1038/nn.2578 20562868

[B105] Patel S. H. Salvi J. D. Ó Maoiléidigh D. Hudspeth A. J. (2012). Frequency-selective exocytosis by ribbon synapses of hair cells in the bullfrog’s amphibian papilla. *J. Neurosci.* 32 13433–13438. 10.1523/JNEUROSCI.1246-12.2012 23015434 PMC3468150

[B106] Peineau T. Belleudy S. Pietropaolo S. Bouleau Y. Dulon D. (2021). Synaptic release potentiation at aging auditory ribbon synapses. *Front. Aging Neurosci.* 13:756449. 10.3389/fnagi.2021.756449 34733152 PMC8558230

[B107] Peulen O. Rademaker G. Anania S. Turtoi A. Bellahcène A. Castronovo V. (2019). Ferlin overview: from membrane to cancer biology. *Cells* 8:E954. 10.3390/cells8090954 31443490 PMC6770723

[B108] Piper A.-K. Ross S. E. Redpath G. M. Lemckert F. A. Woolger N. Bournazos A. (2017). Enzymatic cleavage of myoferlin releases a dual C2-domain module linked to ERK signalling. *Cell Signal* 33 30–40. 10.1016/j.cellsig.2017.02.009 28192161 PMC5995151

[B109] Platzer J. Engel J. Schrott-Fischer A. Stephan K. Bova S. Chen H. (2000). Congenital deafness and sinoatrial node dysfunction in mice lacking class D L-type Ca^2+^ channels. *Cell* 102 89–97. 10.1016/s0092-8674(00)00013-1 10929716

[B110] Ramakrishnan N. A. Drescher M. J. Drescher D. G. (2009). Direct interaction of otoferlin with syntaxin 1A, SNAP-25, and the L-type voltage-gated calcium channel Cav1.3. *J. Biol. Chem.* 284 1364–1372. 10.1074/jbc.M803605200 19004828 PMC2615516

[B111] Ramakrishnan N. A. Drescher M. J. Morley B. J. Kelley P. M. Drescher D. G. (2014). Calcium regulates molecular interactions of otoferlin with soluble NSF attachment protein receptor (SNARE) proteins required for hair cell exocytosis. *J. Biol. Chem.* 289 8750–8766. 10.1074/jbc.M113.480533 24478316 PMC3979417

[B112] Rankovic V. Vogl C. Dörje N. M. Bahader I. Duque-Afonso C. J. Thirumalai A. (2020). Overloaded adeno-associated virus as a novel gene therapeutic tool for otoferlin-related deafness. *Front. Mol. Neurosci.* 13:600051. 10.3389/fnmol.2020.600051 33488357 PMC7817888

[B113] Redpath G. M. I. Sophocleous R. A. Turnbull L. Whitchurch C. B. Cooper S. T. (2016). Ferlins show tissue-specific expression and segregate as plasma membrane/late Endosomal or trans-golgi/recycling Ferlins. *Traffic* 17 245–266. 10.1111/tra.12370 26707827

[B114] Redpath G. M. I. Woolger N. Piper A. K. Lemckert F. A. Lek A. Greer P. A. (2014). Calpain cleavage within dysferlin exon 40a releases a synaptotagmin-like module for membrane repair. *MBoC* 25 3037–3048. 10.1091/mbc.e14-04-0947 25143396 PMC4230592

[B115] Reisinger E. Bresee C. Neef J. Nair R. Reuter K. Bulankina A. (2011). Probing the functional equivalence of otoferlin and synaptotagmin 1 in exocytosis. *J. Neurosci.* 31 4886–4895. 10.1523/JNEUROSCI.5122-10.2011 21451027 PMC3083821

[B116] Ricci A. J. Bai J.-P. Song L. Lv C. Zenisek D. Santos-Sacchi J. (2013). Patch-clamp recordings from lateral line neuromast hair cells of the living zebrafish. *J. Neurosci.* 33 3131–3134. 10.1523/JNEUROSCI.4265-12.2013 23407967 PMC3684625

[B117] Rouillon I. Marcolla A. Roux I. Marlin S. Feldmann D. Couderc R. (2006). Results of cochlear implantation in two children with mutations in the OTOF gene. *Int. J. PediatrOtorhinolaryngol.* 70 689–696. 10.1016/j.ijporl.2005.09.006 16226319

[B118] Roux I. Safieddine S. Nouvian R. Grati M. Simmler M.-C. Bahloul A. (2006). Otoferlin, defective in a human deafness form, is essential for exocytosis at the auditory ribbon synapse. *Cell* 127 277–289. 10.1016/j.cell.2006.08.040 17055430

[B119] Ruel J. Emery S. Nouvian R. Bersot T. Amilhon B. Van Rybroek J. M. (2008). Impairment of SLC17A8 encoding vesicular glutamate transporter-3, VGLUT3, underlies nonsyndromic deafness DFNA25 and inner hair cell dysfunction in null mice. *Am. J. Hum. Genet.* 83 278–292. 10.1016/j.ajhg.2008.07.008 18674745 PMC2495073

[B120] Rutherford M. A. Bhattacharyya A. Xiao M. Cai H.-M. Pal I. Rubio M. E. (2023). GluA3 subunits are required for appropriate assembly of AMPAR GluA2 and GluA4 subunits on cochlear afferent synapses and for presynaptic ribbon modiolar-pillar morphology. *eLife* 12:e80950. 10.7554/eLife.80950 36648432 PMC9891727

[B121] Safieddine S. Wenthold R. J. (1999). SNARE complex at the ribbon synapses of cochlear hair cells: analysis of synaptic vesicle- and synaptic membrane-associated proteins. *Eur. J. Neurosci.* 11 803–812. 10.1046/j.1460-9568.1999.00487.x 10103074

[B122] Schnee M. E. Santos-Sacchi J. Castellano-Muñoz M. Kong J.-H. Ricci A. J. (2011). Calcium-dependent synaptic vesicle trafficking underlies indefatigable release at the hair cell afferent fiber synapse. *Neuron* 70 326–338. 10.1016/j.neuron.2011.01.031 21521617 PMC3254016

[B123] Schug N. Braig C. Zimmermann U. Engel J. Winter H. Ruth P. (2006). Differential expression of otoferlin in brain, vestibular system, immature and mature cochlea of the rat. *Eur. J. Neurosci.* 24 3372–3380. 10.1111/j.1460-9568.2006.05225.x 17229086

[B124] Schwander M. Sczaniecka A. Grillet N. Bailey J. S. Avenarius M. Najmabadi H. (2007). A forward genetics screen in mice identifies recessive deafness traits and reveals that pejvakin is essential for outer hair cell function. *J. Neurosci.* 27 2163–2175. 10.1523/JNEUROSCI.4975-06.2007 17329413 PMC6673480

[B125] Selvakumar D. Drescher M. J. Deckard N. A. Ramakrishnan N. A. Morley B. J. Drescher D. G. (2017). Dopamine D1A directly interacts with otoferlin synaptic pathway proteins: Ca^2 +^ and phosphorylation underlie an NSF-to-AP2mu1 molecular switch. *Biochem. J.* 474 79–104. 10.1042/BCJ20160690 27821621 PMC6310132

[B126] Sharpe H. J. Stevens T. J. Munro S. (2010). A comprehensive comparison of transmembrane domains reveals organelle-specific properties. *Cell* 142 158–169. 10.1016/j.cell.2010.05.037 20603021 PMC2928124

[B127] So H. Kim H. Lee J. Park C. Kim Y. Kim E. (2007). Cisplatin cytotoxicity of auditory cells requires secretions of proinflammatory cytokines via activation of ERK and NF-kappaB. *J. Assoc. Res. Otolaryngol.* 8 338–355. 10.1007/s10162-007-0084-9 17516123 PMC2538433

[B128] Song D. H. Ko G. H. Lee J. H. Lee J. S. Yang J. W. Kim M. H. (2017). Prognostic role of myoferlin expression in patients with clear cell renal cell carcinoma. *Oncotarget* 8 89033–89039. 10.18632/oncotarget.21645 29179496 PMC5687666

[B129] Spassova M. A. Avissar M. Furman A. C. Crumling M. A. Saunders J. C. Parsons T. D. (2004). Evidence that rapid vesicle replenishment of the synaptic ribbon mediates recovery from short-term adaptation at the hair cell afferent synapse. *J. Assoc. Res. Otolaryngol.* 5 376–390. 10.1007/s10162-004-5003-8 15675002 PMC2504567

[B130] Stalmann U. Franke A. J. Al-Moyed H. Strenzke N. Reisinger E. (2021). Otoferlin is required for proper synapse maturation and for maintenance of inner and outer hair cells in mouse models for DFNB9. *Front. Cell Neurosci.* 15:677543. 10.3389/fncel.2021.677543 34335185 PMC8316924

[B131] Stenson P. D. Ball E. V. Mort M. Phillips A. D. Shiel J. A. Thomas N. S. T. (2003). Human gene mutation database (HGMD): 2003 update. *Hum. Mutat.* 21 577–581. 10.1002/humu.10212 12754702

[B132] Stone T. A. Schiller N. von Heijne G. Deber C. M. (2015). Hydrophobic blocks facilitate lipid compatibility and translocon recognition of transmembrane protein sequences. *Biochemistry* 54 1465–1473. 10.1021/bi5014886 25635746 PMC4341838

[B133] Strenzke N. Chakrabarti R. Al-Moyed H. Müller A. Hoch G. Pangrsic T. (2016). Hair cell synaptic dysfunction, auditory fatigue and thermal sensitivity in otoferlin Ile515Thr mutants. *EMBO J.* 35 2519–2535. 10.15252/embj.201694564 27729456 PMC5283603

[B134] Striessnig J. Pinggera A. Kaur G. Bock G. Tuluc P. (2014). L-type Ca^2+^ channels in heart and brain. *Wiley Interdiscip. Rev. Membr. Trans. Signal.* 3 15–38. 10.1002/wmts.102 24683526 PMC3968275

[B135] Sula A. Cole A. R. Yeats C. Orengo C. Keep N. H. (2014). Crystal structures of the human Dysferlin inner DysF domain. *BMC Struc. Biol.* 14:3. 10.1186/1472-6807-14-3 24438169 PMC3898210

[B136] Sutton R. B. Davletov B. A. Berghuis A. M. Südhof T. C. Sprang S. R. (1995). Structure of the first C2 domain of synaptotagmin I: a novel Ca^2+^/phospholipid-binding fold. *Cell* 80 929–938. 10.1016/0092-8674(95)90296-1 7697723

[B137] Tang H. Wang H. Wang S. Hu S. W. Lv J. Xun M. (2022). Hearing of Otof-deficient mice restored by trans-splicing of N- and C-terminal otoferlin. *Hum. Genet.* 142 289–304. 10.1007/s00439-022-02504-2 36383253

[B138] Tertrais M. Bouleau Y. Emptoz A. Belleudy S. Sutton R. B. Petit C. (2019). Viral transfer of mini-Otoferlins partially restores the fast component of exocytosis and uncovers ultrafast endocytosis in auditory hair cells of Otoferlin Knock-out mice. *J. Neurosci.* 39 3394–3411. 10.1523/JNEUROSCI.1550-18.2018 30833506 PMC6495124

[B139] Therrien C. Di Fulvio S. Pickles S. Sinnreich M. (2009). Characterization of lipid binding specificities of dysferlin C2 domains reveals novel interactions with phosphoinositides. *Biochemistry* 48 2377–2384. 10.1021/bi802242r 19253956

[B140] Tong B. Hornak A. J. Maison S. F. Ohlemiller K. K. Liberman M. C. Simmons D. D. (2016). Oncomodulin, an EF-hand Ca^2+^ buffer, is critical for maintaining cochlear function in mice. *J. Neurosci.* 36 1631–1635. 10.1523/JNEUROSCI.3311-15.2016 26843644 PMC4737773

[B141] Tsuzuki N. Namba K. Saegusa C. Mutai H. Nishiyama T. Oishi N. (2023). Apoptosis of type I spiral ganglion neuron cells in Otof-mutant mice. *Neurosci. Lett.* 803 137178. 10.1016/j.neulet.2023.137178 36914046

[B142] Turtoi A. Blomme A. Bellahcène A. Gilles C. Hennequière V. Peixoto P. (2013). Myoferlin is a key regulator of EGFR activity in breast cancer. *Cancer Res.* 73 5438–5448. 10.1158/0008-5472.CAN-13-1142 23864327

[B143] Uthaiah R. C. Hudspeth A. J. (2010). Molecular anatomy of the hair cell’s ribbon synapse. *J. Neurosci.* 30 12387–12399. 10.1523/JNEUROSCI.1014-10.2010 20844134 PMC2945476

[B144] Varga R. Avenarius M. R. Kelley P. M. Keats B. J. Berlin C. I. Hood L. J. (2006). OTOF mutations revealed by genetic analysis of hearing loss families including a potential temperature sensitive auditory neuropathy allele. *J. Med. Genet.* 43 576–581. 10.1136/jmg.2005.038612 16371502 PMC2593030

[B145] Vincent P. (2015). *The spatial organization of Cav1.3 calcium channels determines the efficiency of ribbon synapse exocytosis in inner ear hair cells.* Available online at: https://theses.hal.science/tel-01969394 (accessed March 29, 2023).

[B146] Vincent P. F. Bouleau Y. Petit C. Dulon D. (2015). A synaptic F-actin network controls otoferlin-dependent exocytosis in auditory inner hair cells. *eLife* 4:e10988. 10.7554/eLife.10988 26568308 PMC4714970

[B147] Vincent P. F. Y. Bouleau Y. Charpentier G. Emptoz A. Safieddine S. Petit C. (2017). Different CaV1.3 channel isoforms control distinct components of the synaptic vesicle cycle in auditory inner hair cells. *J. Neurosci.* 37 2960–2975. 10.1523/JNEUROSCI.2374-16.2017 28193694 PMC6596729

[B148] Vincent P. F. Y. Bouleau Y. Safieddine S. Petit C. Dulon D. (2014). Exocytotic machineries of vestibular type I and cochlear ribbon synapses display similar intrinsic otoferlin-dependent Ca^2+^ sensitivity but a different coupling to Ca^2+^ channels. *J. Neurosci.* 34 10853–10869. 10.1523/JNEUROSCI.0947-14.2014 25122888 PMC6705247

[B149] Vincent P. F. Y. Cho S. Tertrais M. Bouleau Y. von Gersdorff H. Dulon D. (2018). Clustered Ca^2+^ channels are blocked by synaptic vesicle proton release at mammalian auditory ribbon synapses. *Cell Rep.* 25 3451.e3–3464.e3. 10.1016/j.celrep.2018.11.072 30566869 PMC6365105

[B150] Vogl C. Cooper B. H. Neef J. Wojcik S. M. Reim K. Reisinger E. (2015). Unconventional molecular regulation of synaptic vesicle replenishment in cochlear inner hair cells. *J. Cell Sci.* 128 638–644. 10.1242/jcs.162099 25609709

[B151] Vogl C. Panou I. Yamanbaeva G. Wichmann C. Mangosing S. J. Vilardi F. (2016). Tryptophan-rich basic protein (WRB) mediates insertion of the tail-anchored protein otoferlin and is required for hair cell exocytosis and hearing. *EMBO J.* 35 2536–2552. 10.15252/embj.201593565 27458190 PMC5283584

[B152] Vona B. Rad A. Reisinger E. (2020). The many faces of DFNB9: relating OTOF variants to hearing impairment. *Genes* 11:E1411. 10.3390/genes11121411 33256196 PMC7768390

[B153] Wakabayashi K. Fujioka M. Kanzaki S. Okano H. Shibata S. Yamashita D. (2010). Blockade of interleukin-6 signaling suppressed cochlear inflammatory response and improved hearing impairment in noise-damaged mice cochlea. *Neurosci. Res.* 66 345–352. 10.1016/j.neures.2009.12.008 20026135

[B154] Wang G. Galli T. (2018). Reciprocal link between cell biomechanics and exocytosis. *Traffic* 19 741–749. 10.1111/tra.12584 29943478

[B155] Wang T. Xu F. Huo Y. Potier-Ferry M. (2018). Snap-through instabilities of pressurized balloons: pear-shaped bifurcation and localized bulging. *Int. J. Non Linear Mech.* 98 137–144. 10.1016/j.ijnonlinmec.2017.10.017

[B156] Wang W.-S. Liu X.-H. Liu L.-X. Lou W.-H. Jin D.-Y. Yang P.-Y. (2013). iTRAQ-based quantitative proteomics reveals myoferlin as a novel prognostic predictor in pancreatic adenocarcinoma. *J. Proteomics* 91 453–465. 10.1016/j.jprot.2013.06.032 23851313

[B157] Wittig J. H. Parsons T. D. (2008). Synaptic ribbon enables temporal precision of hair cell afferent synapse by increasing the number of readily releasable vesicles: a modeling study. *J. Neurophysiol.* 100 1724–1739. 10.1152/jn.90322.2008 18667546 PMC2576205

[B158] Xu L. Pallikkuth S. Hou Z. Mignery G. Robia S. Han R. (2011). Dysferlin forms a dimer mediated by the C2 domains and the transmembrane domain *in vitro* and in living cells. *PLoS One* 6:e27884. 10.1371/journal.pone.0027884 22110769 PMC3215728

[B159] Xue M. Ma C. Craig T. K. Rosenmund C. Rizo J. (2008). The janus-faced nature of the C(2)B domain is fundamental for synaptotagmin-1 function. *Nat. Struct. Mol. Biol.* 15 1160–1168. 10.1038/nsmb.1508 18953334 PMC2587052

[B160] Yadav A. Kumar B. Lang J. C. Teknos T. N. Kumar P. (2017). A muscle-specific protein “myoferlin” modulates IL-6/STAT3 signaling by chaperoning activated STAT3 to nucleus. *Oncogene* 36 6374–6382. 10.1038/onc.2017.245 28745314 PMC5690845

[B161] Yasunaga S. Grati M. Chardenoux S. Smith T. N. Friedman T. B. Lalwani A. K. (2000). OTOF encodes multiple long and short isoforms: genetic evidence that the long ones underlie recessive deafness DFNB9. *Am. J. Hum. Genet.* 67 591–600. 10.1086/303049 10903124 PMC1287519

[B162] Yasunaga S. Grati M. Cohen-Salmon M. El-Amraoui A. Mustapha M. Salem N. (1999). A mutation in OTOF, encoding otoferlin, a FER-1-like protein, causes DFNB9, a nonsyndromic form of deafness. *Nat. Genet.* 21 363–369. 10.1038/7693 10192385

[B163] Yin Y. Henzl M. T. Lorber B. Nakazawa T. Thomas T. T. Jiang F. (2006). Oncomodulin is a macrophage-derived signal for axon regeneration in retinal ganglion cells. *Nat. Neurosci.* 9 843–852. 10.1038/nn1701 16699509

[B164] You Z. Ge A. Pang D. Zhao Y. Xu S. (2020). Long noncoding RNA FER1L4 acts as an oncogenic driver in human pan-cancer. *J. Cell Physiol.* 235 1795–1807. 10.1002/jcp.29098 31332783

[B165] Zak M. Bress A. Brandt N. Franz C. Ruth P. Pfister M. (2012). Ergic2, a brain specific interacting partner of otoferlin. *Cell Physiol. Biochem.* 29 941–948. 10.1159/000188338 22613993

[B166] Zhang Q.-J. Han B. Lan L. Zong L. Shi W. Wang H.-Y. (2016). High frequency of OTOF mutations in Chinese infants with congenital auditory neuropathy spectrum disorder. *Clin Genet.* 90 238–246. 10.1111/cge.12744 26818607

[B167] Zhang T. Li J. He Y. Yang F. Hao Y. Jin W. (2018). A small molecule targeting myoferlin exerts promising anti-tumor effects on breast cancer. *Nat. Commun.* 9:3726. 10.1038/s41467-018-06179-0 30213946 PMC6137146

[B168] Zheng D. Liu X. (2020). Cochlear implantation outcomes in patients with OTOF mutations. *Front. Neurosci.* 14:447. 10.3389/fnins.2020.00447 32508568 PMC7253664

[B169] Zhu Y. Li Q. Gao X. Li Y. Liu Y. Dai P. (2021). Familial temperature-sensitive auditory neuropathy: distinctive clinical courses caused by variants of the *OTOF* gene. *Front. Cell Dev. Biol.* 9:732930. 10.3389/fcell.2021.732930 34692690 PMC8529165

[B170] Zwicker E. (1965). Temporal effects in simultaneously masking and loudness. *J. Acoust. Soc. Am.* 38 132–141. 10.1121/1.1909588 14347604

